# Advanced mycotoxin control and decontamination techniques in view of an increased aflatoxin risk in Europe due to climate change

**DOI:** 10.3389/fmicb.2022.1085891

**Published:** 2023-01-10

**Authors:** Martina Loi, Antonio F. Logrieco, Tünde Pusztahelyi, Éva Leiter, László Hornok, István Pócsi

**Affiliations:** ^1^Institute of Sciences of Food Production, National Research Council, Bari, Italy; ^2^Central Laboratory of Agricultural and Food Products, Faculty of Agricultural and Food Sciences and Environmental Management, University of Debrecen, Debrecen, Hungary; ^3^Department of Molecular Biotechnology and Microbiology, Faculty of Science and Technology, Institute of Biotechnology, University of Debrecen, Debrecen, Hungary; ^4^ELRN-UD Fungal Stress Biology Research Group, University of Debrecen, Debrecen, Hungary; ^5^Hungarian University of Agriculture and Life Sciences, Gödöllő, Hungary

**Keywords:** aflatoxin, climate change, models, mycotoxin control, decontamination techniques

## Abstract

Aflatoxins are toxic secondary metabolites produced by *Aspergillus* spp. found in staple food and feed commodities worldwide. Aflatoxins are carcinogenic, teratogenic, and mutagenic, and pose a serious threat to the health of both humans and animals. The global economy and trade are significantly affected as well. Various models and datasets related to aflatoxins in maize have been developed and used but have not yet been linked. The prevention of crop loss due to aflatoxin contamination is complex and challenging. Hence, the set-up of advanced decontamination is crucial to cope with the challenge of climate change, growing population, unstable political scenarios, and food security problems also in European countries. After harvest, decontamination methods can be applied during transport, storage, or processing, but their application for aflatoxin reduction is still limited. Therefore, this review aims to investigate the effects of environmental factors on aflatoxin production because of climate change and to critically discuss the present-day and novel decontamination techniques to unravel gaps and limitations to propose them as a tool to tackle an increased aflatoxin risk in Europe.

## 1. Introduction

Mycotoxins are toxic secondary metabolites produced by filamentous fungi which contaminate food and feed products worldwide. More than 25% of food is contaminated by at least one mycotoxin, with important implications for the health of humans and animals and the global trade and economy (Eskola et al., 2020; Ráduly et al., 2020).

Aflatoxins (AFs) are the most critical mycotoxins due to their toxic potential and occurrence. Indeed, they have been listed as Class 1 carcinogens by the International Agency on Research on Cancer, known to cause cancer, namely liver cancer, in humans (International Agency for Research on Cancer, 2002). Besides, they may cause liver inflammation and necrosis, immune depression, stunting, growth and development impairment, reproductive dysfunction, and even death when consumed at high dosages (Rushing and Selim, 2019; Ráduly et al., 2020).

AFs include more than 20 different furanocoumarin derivatives, which can be found on a wide variety of food commodities in the field during harvest, transport, and storage (Kumar et al., 2017). The most relevant ones are the AFs of the B-series (AFB_1_ and AFB_2_) and G-series (AFG_1_ and AFG_2_), which can be found in cereals, peanuts, nuts, and spices, and AFs of the M-series (AFM_1_ and AFM_2_), which are *in vivo* hydroxylated metabolites secreted in milk.

Being secondary metabolites, the production of AFs is sophistically regulated and depends upon a wide spectrum of biotic and abiotic factors (Reverberi et al., 2010; Bayram and Braus, 2012; Fountain et al., 2014; Keller, 2015; Pfliegler et al., 2020; Khan et al., 2021), thus making it difficult to prevent entirely. Pre-harvest technologies mainly rely on good agricultural practices and the field application of non-aflatoxigenic fungal strains as biological control agents displacing aflatoxigenic strains on crops (Abbas et al., 2017; Agbetiameh et al., 2019; Sarrocco et al., 2019; Dövényi-Nagy et al., 2020; Peles et al., 2021; Ola et al., 2022).

Once synthesized, AFs persist in food and feed because they are highly stable to heat and most common food and feed processing techniques. To reduce AFs contamination, various decontamination methods can be applied after harvest, either during transport, storage, or processing (Peles et al., 2021; Sipos et al., 2021). Considering that commodities can be imported from distant geographical areas and stored for long periods before being processed, all post-harvest handling stages are important to reduce aflatoxin contamination.

Cereals like maize, sorghum, wheat, and barley are amongst the most susceptible commodities to be contaminated by AFs. They represent the staple food of human and animal diet worldwide and will be increasingly required to feed the growing population. Nonetheless, cereal trade globalization is threatened not only by fungal infection and mycotoxin contamination but also by climate change and insecure political scenarios (King et al., 2017; Sarmast et al., 2021; Hellegers, 2022).

Cereal production is expected to decrease slightly in 2022/2023, making it an even more precious commodity in the following years (FAO, 2022). In Europe, the production in 2021 has been estimated to be around 270 million tons (FAOstat, 2022) and it will be 4% lower due to hot and dry weather conditions, prolonged periods of rainfall deficits, and reduced maize output caused by the war in Ukraine (FAO, 2022). Besides tighter supplies and market uncertainty, rising energy and input prices will also contribute to keeping world cereal prices elevated throughout 2023 (FAO, 2022). To further complicate this scenario, in the near future, climate change will significantly modify fungal and mycotoxin contamination patterns, and aflatoxin contamination of maize will be the main mycotoxin issue in Europe (Moretti et al., 2019). Aside from the increased contamination in the fields, increased aflatoxin M_1_ levels (also above the current European regulation limit of 50 ng/kg) are also expected in milk and dairy (Van der Fels-Klerx et al., 2019).

**Figure 1:**
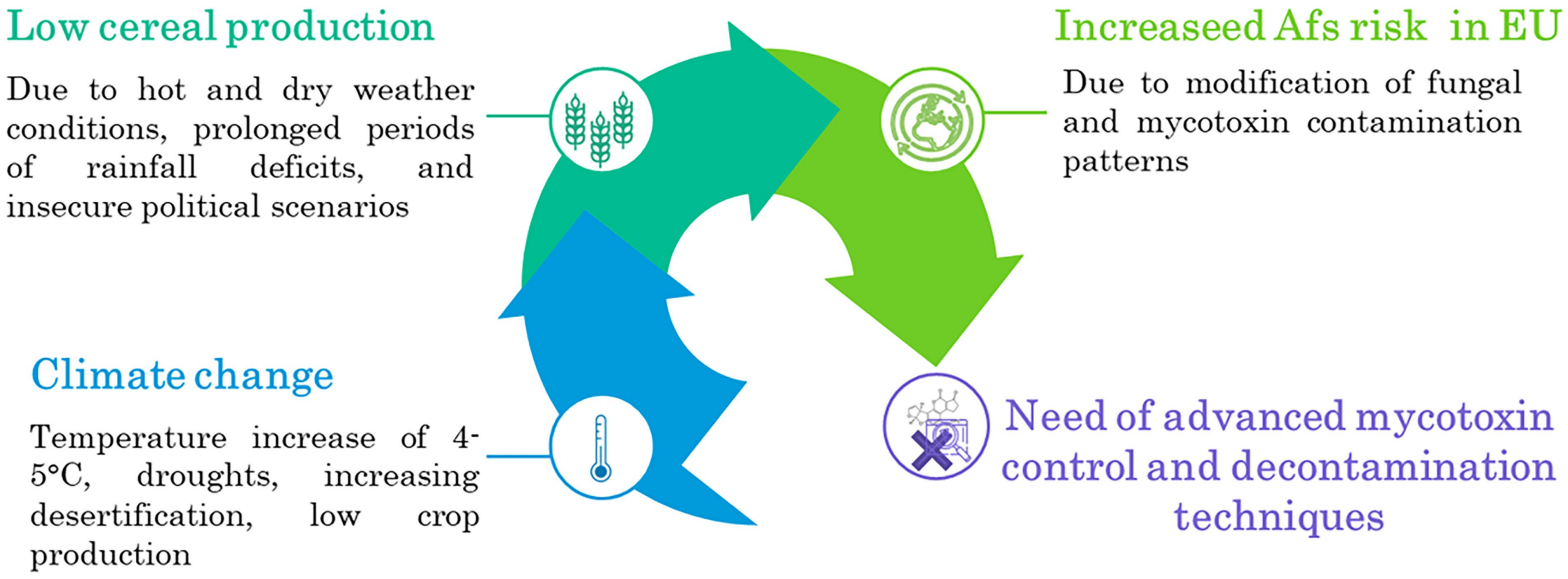
Predicted processes in a climate change scenario.

Nuts and nut products and oilseed are other two important AFs susceptible commodities. Nontheless, European production of these two commodities is far behind that of cereals, namely 2 and 27 million tons, respectively (FAOstat, 2022). While nuts are widely imported from abroad (e.g., Turkey, United States, Iran, Vietnam, China), they remain the most notified product category contaminated by AFs in 2021 by the Rapid Alert System for Food and Feed (2022). Regarding oilseed production, The EU cultivates three major types of oilseed crops; the main three are rape, sunflower, and soya. From a safety point of view, they do not represent the major oilseed crops to be contaminated by AFs (Einolghozati et al., 2021).Therefore, the set-up and application of advanced decontamination are crucial to cope with the challenge of climate change, growing population, unstable political scenarios, and food security problems, as summarized in Figure 1.

Due to the aflatoxin worldwide concern, over the past 20 years a wide variety of decontamination methods have been investigated, including physical, chemical, and biological ones. Therefore, this review aims to investigate the effects of environmental factors on aflatoxin production in view of the climate change to better understand how to model and prevent contamination in the field, as well as the present-day and novel decontamination techniques which can be applied to the in the field and at post-harvest stage. Gaps and perspectives of these methods were also critically discussed to propose them as a tool to tackle an increased aflatoxin risk in Europe.

## 2. Genetics of aflatoxin production

Molecular genetic tools are absolutely needed (i) to decipher the function of aflatoxin biosynthetic genes (Amaike and Keller, 2011; Amare and Keller, 2014; Caceres et al., 2020; Gil-Serna et al., 2020), (ii) to shed light on the regulatory motifs and networks governing this high-complexity process (Yu, 2012; Eom et al., 2018; Pfannenstiel et al., 2018; Cary et al., 2019; Gil-Serna et al., 2020; Zhao et al., 2022), (iii) to track back genetic changes resulting in non-aflatoxigenic biological control fungal strains (Moore et al., 2009; Chang et al., 2012a; Adhikari et al., 2016; Pennerman et al., 2018; Chang, 2022), (iv) to study population structure and dynamics of aflatoxigenic molds in agriculture (Chang et al. 2019; Lewis et al., 2019; Weaver et al., 2019; Drott et al., 2020), (v) to map the mechanism of action of various agents interfering with the aflatoxin production by aflatoxigenic molds (Hua et al., 2014; Wang et al., 2015; Ren Y. et al., 2020; Safari et al., 2020), and (vi) to develop novel, RNA interference-based, host-induced AF control technologies (Majumdar et al., 2017; Wu, 2022). To make genetics and biotechnology-based approaches and innovations more effective, we need to intensify basic research aiming at the elucidation of the elements of the regulatory network that fine-tunes aflatoxin production adequately to the environmental stimuli perceived by aflatoxin producer molds.

The organization of the aflatoxin biosynthetic gene cluster and the functions of its genes have recently been reviewed in several publications (Caceres et al., 2020; Gil-Serna et al., 2020; Khan et al., 2021; Ferrara et al., 2022), and, therefore, only a short recapitulation of our knowledge on the genetic regulation of AF biosynthesis is presented here.

The biosynthesis of AFs is a complex, energy-consuming process that needs, as a minimum, 27 enzymatic reactions to build up these highly complex molecules. Studies on the genetic background of AF biosynthesis in *A. flavus* and *A. parasiticus*, as well as sterigmatocystin production in *A. nidulans*, led to the identification of the AF gene cluster (Yu et al., 2000, 2004; Georgianna and Payne, 2009; Amare and Keller, 2014).

The 75 kbp long AF gene cluster comprises 30 genes and is located near these fungi’s telomere of chromosome III. Of the enzyme encoding structural genes of the cluster, four genes, *aflA*, *aflB*, *aflC*, and *hypC*, encoding two fatty acid synthase subunits, a polyketide synthase, and an oxidase, respectively, are involved in building up the precursor, norsoloric acid (NOR) from hexanoate units. NOR is converted to versicolorin B (VERB) by a series of enzymes encoded by *aflD*, *aflG*, *aflH*, *aflI*, *aflJ*, *aflK*, *aflV*, and *aflW* genes. Transformation of VERB into versicolorin A (VERA) is catalyzed by a cytochrome P-450 monooxygenase, encoded by *aflL*. Five genes (*aflM*, *aflN*, *aflY*, *aflX*, and *aflO*) are responsible for converting VERA to sterigmatocystin (STER). Finally, STER is transformed into AFB_1_ in several *Aspergillus* species belonging to section *Flavi* with the aid of enzymes encoded by *aflP*, *aflQ*, *hypB*, *hypE*, and *aflE*. An additional gene of the cluster (*aflT*), encoding a putative MFS transporter, was found to have no significant role in AF secretion (Chang et al., 2004), despite the presence of its gene product (AflT) in the aflatoxisomes (Chanda et al., 2010).

The central positive regulator of the AF biosynthesis genes is *aflR* (Yu et al., 1996), encoding a Zn(II)2Cys6 transcription factor (Shimizu et al., 2003). The AflR protein, acting as a pathway-specific transcription factor, has binding sites on promoters of as many as 18 *afl* genes, including its own promoter, indicating autoregulation of *aflR* (Chang et al., 1995). Within the cluster, next to *aflR*, there is another regulatory gene, *aflS* acting as an enhancer. The product of this gene interacts with AflR, and the AflR-AflS complex formed in this manner allows a stringent binding to promoters of the target genes (Kong et al., 2014).

Not surprisingly, a wide spectrum of RNA interference based technologies has been developed and tested *in planta* to control AF production by aflatoxigenic fungi (McDonald et al., 2005;Majumdar et al., 2017; Wu, 2022). A frequently targeted gene is *aflR* in both peanut (Arias et al., 2015; Faustinelli et al., 2018; Power et al., 2020) and maize (Masanga et al., 2015). Other approaches targeted either the *aflS* regulatory gene (Arias et al., 2015; Power et al., 2020) or AF biosynthetic genes including *aflC* (encoding polyketide synthase; Arias et al., 2015; Thakare et al., 2017; Power et al., 2020; Niño-Sánchez et al., 2021) and *aflM* (coding for versicolorin dehydrogenase; Raruang et al., 2020). It is noteworthy that a RNAi-based AF control system simultaneously silencing *aflR*, *aflR*, *aflC*, *aflep* (coding for AF efflux pump) and *pes1* (encoding a non-ribosomal peptide synthase with hypothesized function in cyclopiazonic acid biosynthesis) was also constructed and tested in peanut using both transgenic and non-transgenic delivery tools (Arias et al., 2015; Power et al., 2020). In addition to host induced gene silencing strategies (Majumdar et al., 2017; Wu, 2022), exogenous RNAi delivery-based, non-transgenic approaches are gaining ground including the application of RNAi-triggering dsDNA and dsRNA by gene gun (Power et al., 2020), DsiRNA (Dicer-substrate siRNA) after wounding (Faustinelli et al., 2018) and dsRNA by genetically engineered bacteria (RNAseIII-null mutant *Escherichia coli*, both living cells and crude whole-cell autolysates; Niño-Sánchez et al., 2021). Further AF biosynthetic structural genes like *aflD* (encoding an enzyme converting norsolorinic acid to averantin; Abdel-Hadi et al., 2011) are likely to be targeted in future RNA interference technologies.

In addition to pathway-specific transcription factors, secondary metabolite biosynthesis genes are regulated by global (or general) transcription factors, too; these transcription factors mediate (i) nutritional, (ii) environmental, and (iii) developmental signals (Reverberi et al., 2010; Roze et al., 2011; Yin et al., 2012, 2013; Montibus et al., 2013; Hong et al., 2013a,b; Ferrara et al., 2022). Such a complex regulatory network helps fungi to react to stressors by producing secondary metabolites. For example, AF biosynthesis has long been recognized as an essential oxidative stress response mechanism in *Aspergilli* (Roze et al., 2011), and AFs are natural scavengers of reactive oxygen species (Finotti et al., 2021).

As far as the nutrition-related factors are concerned, CreA and AreA are worth mentioning first. CreA, encoded by *creA*, a Cys2His2 zinc finger transcription factor, is the critical player in carbon catabolite repression in filamentous fungi. Deletion of *creA* in *A. flavus* resulted in the loss of AF production (Fasoyin et al., 2018), indicating a crucial role of this TF in regulating the AF gene cluster. Another transcription factor, AreA, a member of the GATA transcription factor family and acting as the primary regulator of N-utilization, also affects AF biosynthesis. A *ΔareA* strain of *A. flavus* increased or decreased AF production compared to the wild type depending on the N-source of the culture medium (Fasoyin et al., 2019). Among nitrogen forms, nitrate inhibits AF formation, increases the expression of *aflS* gene, and reduces the expression of other genes involved in AF synthesis (Price et al., 2005). However, actual AF synthesis is inhibited by a pathway other than AF synthesis, which is hypothesized that is associated with a change in the redox potential caused by nitrate, which affects the formation of the precursor of polyketide synthesis by increasing the activity of mannitol dehydrogenase (Niehaus and Jiang, 1989).

Simple sugars and acetate stimulate (Shantha and Murthy, 1981), and intermediates of the tricarboxylic acid cycle inhibit the formation of AF (Buchanan and Ayres, 1977; Shantha and Murthy, 1981). Although, at the same time, carbon sources do not regulate specifically the genes in the AF biosynthesis pathway. They affect the synthesis of AFs either through the precursors or the cAMP signaling pathway (Georgianna and Payne, 2009).

Further studies on the regulatory network fine-tuning the carbon and nitrogen metabolisms of aflatoxigenic molds are definitely needed because host-induced gene silencing of either *A. flavus amy1* (encoding alpha-amylase) or *alk* (coding for alkaline protease) significantly reduced growth and AF production (Gilbert et al., 2018; Omolehin et al., 2021). Shedding light on the elements of this network and their target genes may help us to find novel targets for the construction of crops with enhanced AF resistance. Importantly, direct targeting of *A. flavus* by RNAi technologies may be more effective than the suppression of the AF biosynthetic gene cluster especially in late maturing grain under humid conditions (Gressel and Polturak, 2018). Hence, RNA interference-based targeting of other important genes maintaining fungal growth like *spds* (coding for spermidine synthase; Majumdar et al., 2018) is foreseeable.

Transcription factors involved in responses to environmental variables, like pH, oxidative stress, and light, influence AF/STER production and transcription of AF genes. PacC, a Cys2His2 zinc finger regulator, is the critical player in reacting to pH. An *A. nidulans* mutant with constitutive *pacC* activity produced 10-fold less STER than its wild-type parental strain (Keller et al., 1997). Acidic pH favors AF synthesis through the transcription factor PacC, whose binding point is in the promoter of *aflR*, while the inhibitory effect of alkaline pH on AF synthesis is realized through *pkA* (Tilburn et al., 1995; Ehrlich et al., 1999; Shimizu and Keller, 2001; Shimizu et al., 2003).

AP-1 and AtfB, bZIP transcription factors, MsnA, a Cys2His2 zinc finger, and SrrA, a winged helix-turn-helix transcription factor, were shown to form a regulatory network to mediate oxidative stress response and induce AF biosynthesis in *A. parasiticus* (Hong et al., 2013b). In this fungus, AtfB, by forming a heterodimer with AP-1, binds to seven genes of the AF gene clusters at the CRE binding sites and induces AF biosynthesis (Roze et al., 2011). Therefore, AtfB silencing brought about a remarkable reduction in AF production (Wee et al., 2017). AtfB binding and induction of AF production can be initiated by the elevation of extra- or intracellular ROS (Roze et al., 2011).

A comprehensive study by Zhao et al. (2022) revealed several basic region/leucine zipper motif (bZIPs) transcription factors involved in AF production in *A. flavus*. Ten bZIPs seem to regulate AF biosynthesis since gene deletion of the *bZIP1*, *bZIP2*, *bZIP4*, *bZIP5*, *atfA*, *atfB*, *meaB*, and *metR* reduced AF levels remarkably. It is worth noting that the deletion of *A. nidulans atfA* and *Fusarium verticillioides FvatfA* also resulted in drastically decreased mycotoxin, namely sterigmatocystin and fumonisin productions in these fungi (Szabó et al., 2020; Kocsis et al., 2022). In *A. flavus*, the elimination of the bZIP transcription factors *hapX* and *jlbA* decreased AF biosynthesis slightly but significantly (Zhao et al., 2022). Furthermore, all 10 bZIPs, except bZIP5, coupled with AF production reacted to oxidative stress (Zhao et al., 2022).

The bZIP transcription factor, AflRsmA (restorer of secondary metabolism A) is also associated with the AF production in *A. flavus*. The overexpression of *AflrsmA* increased the AFB_1_ production. At the same time, oxidative stress (menadione sodium bisulfite and *tert*-butyl-hydroperoxide) exposed *ΔAflRsmA* mutant showed reduced AF levels compared to the wild type and *AflRsmA^OE^* strains (Wang et al., 2020). Furthermore, this research group identified conserved motifs in promoters of both AF biosynthesis genes and stress-response genes, where these transcription factors can bind. Interestingly, deletion of Afap1, a *Saccharomyces cerevisiae* Yap1 ortholog bZIP type transcription factor, led to down-regulation of *aflM* and *aflP* and up-regulation of *aflB* and *aflR*, and this contrasting action resulted in a ~ 75% decrease of AF production in *A. flavus* (Guan et al., 2019).

Similar to the regulatory network orchestrating the carbon and nitrogen metabolisms of aflatoxigenic fungi, further elements of the environmental stress response system and their regulation should be revealed and considered as future targets to control the growth and AF production of these molds. Actually, there is a plethora of literature data demonstrating the efficiency of various antioxidants to hinder the AF production by aflatoxigenic fungi (Reverberi et al., 2005, 2006; Zjalic et al., 2006; Caceres et al., 2017; Zhao et al., 2018; Xu et al., 2021). Obviously, a deeper understanding of the regulatory elements interweaving the pathways responsible for the proper adjustment and co-regulation of mycotoxin production and environmental stress response may also help us to develop novel technologies for mycotoxin contamination control.

The light-responsive Velvet complex regulates secondary metabolite genes in a temperature-dependent manner (Lind et al., 2016). Accordingly, components of this complex (VeA, VelB, LaeA) affect AF production. In *A. flavus*, deletion of *laeA* resulted in the downregulation of *aflR*, *aflS*, and *aflD*, accompanied by loss of AF production (Chang et al., 2012b). Whereas, in the *ΔveA* mutants of *A. parasiticus*, no *aflR*, *aflS*, *aflC* and *aflM* transcripts were observed, and the mutant did not produce the AF precursor, versicolorin A (Calvo et al., 2004). VeA of *A. flavus* regulates not only the formation of AFs but also the biosynthesis of cyclopiazonic acid and aflatrem (Duran et al., 2007). Concomitantly, VeA is also a key player in the regulation of H_2_O_2_ stress response in *A. flavus* (Baidya et al., 2014). Interestingly, mycovirus-dependent suppression of AF production by *A. flavus* may also be a result of the interference of mycovirus (*PcV*) degradation products with *veA* expression (Schmidt, 2009). Mycovirus-based technologies to control aflatoxigenic molds can be promising but further molecular-level studies are needed in this field (Kotta-Loizou and Coutts, 2017).

Lipid synthesis correlates with AF production since the first step produces acetyl coenzyme A, which is necessary for forming STER and thus AFs (Dutton, 1988). Oxylipins are signal molecules derived from fatty acids and play an essential role in regulating development, pathogenic processes, and the production of secondary metabolites (Tsitsigiannis and Keller, 2006, 2007). Genes that encode fatty acid oxygenases (*ppoA*, *ppoB*, *ppoC*) affect the expression of *brlA* and *veA* genes; the *veA* gene also regulates the *brlA* gene that affects asexual reproduction, which may have an effect that may be exerted through oxylipins (Tsitsigiannis et al., 2005; Calvo, 2008). The effects of *ppo* genes on *aflR* can be exerted through the PKA or the pathway mediated by the G protein (Tsitsigiannis and Keller, 2006).

Finally, development-related transcription factors can also regulate AF biosynthesis genes. Home-box proteins (Hbx) control basic developmental processes, like conidiogenesis and fruiting body development in fungi. Disruption of *hbx1* in *A. flavus* resulted in the loss of AF production. Transcriptome analysis revealed that Hbx1 is a master regulator, as its deletion affected the expression of more than 5,000 genes in the *A. flavus* genome, including down-regulation of *aflO*, *aflP*, and *aflR* (Cary et al., 2019). NsdC, a Cys2His2 zinc finger and NsdD, a GATA type TF, are required for sexual and asexual development in *Aspergillus* spp. and influence AF biosynthesis. In a *ΔnsdC* mutant of *A. flavus*, transcript levels of *aflR* increased, but that of *aflM* and *aflP* decreased, resulting in a loss of AF production (Cary et al., 2012).

Besides their roles in nucleosome positioning and RNA polymerase recruiting, the global transcription factors we summed up above assist, thus, the AF-producing *Aspergilli* by interconnecting the perception of environmental/developmental signals and the fine regulation of the AF biosynthesis genes. Future studies will hopefully point at novel targets for the development of effective RNA interference-based, host-induced plant biotechnological methods to control AF production in important crops sensitive to aflatoxigenic molds, possibly even in Europe.

## 3. Environmental factors affecting AF production

A proper mapping of environmental factors affecting AF production are of paramount importance when good agricultural practices and effective storage protocols are adapted, developed, and evaluated (Dövényi-Nagy et al., 2020; Sipos et al., 2021). Monitoring these environmental factors may help experts to control AFs in feed and food production chains (Farkas et al., 2022).

Not surprisingly, several environmental factors affect the biosynthesis of AFs, such as temperature (O’Brian et al., 2007), light, pH, carbon sources, and nitrogen sources (Calvo et al., 2002; Price et al., 2005).

Water activity (a*_w_*) and temperature are crucial environmental aspects affecting *A. flavus* growth and AF production. The effect of temperature on AF synthesis is temporary; above the optimal temperature required for synthesis, the genes of the AF gene cluster are repressed, except for *aflR* and *aflS*, the level of which remains at a constant value (O’Brian et al., 2007). However, the level of the AflR protein is much lower at a higher temperature, which can be explained by transcription inhibition of the genes of the AF gene cluster and the inactivation of proteins (Liu and Chu, 1998).

The a*_w_* × temperature connections are related to the ratio of the two essential regulatory genes (*aflS*/*aflR*). The higher ratio of *aflS*/*aflR* relate to the higher level of AFs production (Schmidt-Heydt et al., 2009, 2010; Abdel-Hadi et al., 2010, 2012; Medina et al., 2014). For example, AFB_1_ production on polished rice occurs over a broader range of temperature × a_w_ levels. For fungal growth on polished rice, the optimal conditions were a*_w_* 0.92–0.96 and 28–37°C. The maximum amounts of AFB_1_ were observed at 33°C and a*_w_* 0.96 on polished rice (Lv et al., 2019). Two regulatory genes (*aflR* and *aflS*) were up-regulated at a*_w_* 0.90 (Lv et al., 2019). In addition, the expression of 11 development-related genes amplified under 0.99 a*_w_* treatment (Zhang et al., 2014).

Moreover, the effect of relating conditions of a*_w_* × temperature × elevated CO_2_ had little consequence on fungal growth; they had a significant influence on structural *aflD* and regulatory *aflR* genes and can significantly stimulate the production of AFB_1_ (Medina et al., 2014).

For an *A. nomius* strain, isolated from Brazil nuts, the ideal temperature for growth was 30°C and the best state for expressing the *aflR*, *aflD*, and *aflQ* genes. However, maximum production of AF B and G occurred at 25°C (Yunes et al., 2020). On the other hand, in *A. flavus*, compared with 37°C, the transcript abundance of 30 AFs biosynthesis genes was much higher at 30°C, and most genes were up-regulated at both protein and transcription levels at 28°C (Bai et al., 2015).

Drought stress has been revealed to stimulate the production of reactive oxygen species (ROS) in plant tissues (Cruz de Carvalho, 2008). Cells use their antioxidant system to protect themselves from reactive oxygen radicals, which can react with DNA, proteins and lipids and damage their functions. When the balance between antioxidants and reactive oxygen radicals is lost, oxidative stress occurs (Apel and Hirt, 2004). Oxidative stress stimulates AF synthesis, while antioxidants such as gallic acid adversely affect the formation of AF by inhibiting *aflM* and *aflD* genes (Jayashree and Subramanyam, 2000; Mahoney and Molyneux, 2004; Reverberi et al., 2008). Superoxide dismutase plays an essential role in the effect of oxidative stress on AF formation, which catalyzes the conversion of superoxide into hydrogen peroxide and water and inhibits the formation of AF (Furukawa and Sakuda, 2019). Enzymes involved in oxidative stress presented significantly down-regulated in liquid media and up-regulated at 28°C (*p* ≤ 0.05) in *A. flavus* (Wang et al., 2019).

A clear understanding of how abiotic factors impact aflatoxin contamination is crucial to understand their real impact in new geographical locations in the climate change scenario. This information can be integrated in new models which can be exploited for early prediction (as described in section “Climatic effects and climatic models in Europe”) and pro-active intervention. Today, precision agriculture, nanotechnology, machine learning, and artificial intelligence can be used to set-up an innovative crop management/intervention system with real-time monitoring and responsiveness, especially in controlled-environment agriculture and during storage.

## 4. Climatic effects and climatic models in Europe

Naturally, *A. flavus* is hosted by a wide range of plants, while AF production is most common in plant types with higher oil content (*e.g.*, maize, hazelnut, and other nuts). The distribution and growth of fungi in the soil are altered by geographical areas, soil type, soil water retention, climatic conditions (temperature, humidity, and rainfall), altitude, landform, type of crop grown, rotation, crop, and insect presence (Zhang et al., 2017). In addition, larger population densities were associated with soils with a significant organic matter content, abundant nitrate, phosphate, potassium, increased pH, and more significant electrical conductivity. Fungal spores can be spread by direct contact with the soil, dust-carrying soil particles, or insect vectors (Abbas et al., 2017; Zhang et al., 2017). Soil (specifically agricultural soils), where mould-infected plant residues are often present, serves as the main pool of mycotoxigenic fungi (Zhang et al., 2017). Therefore, considering mycotoxin concentrations, geographical location significantly affects the distribution of AFs. Therefore, the risk of a shift in traditional occurrence areas for AFs is expected in the World, while the incidence of AF is unpredictable.

**Figure 2:**
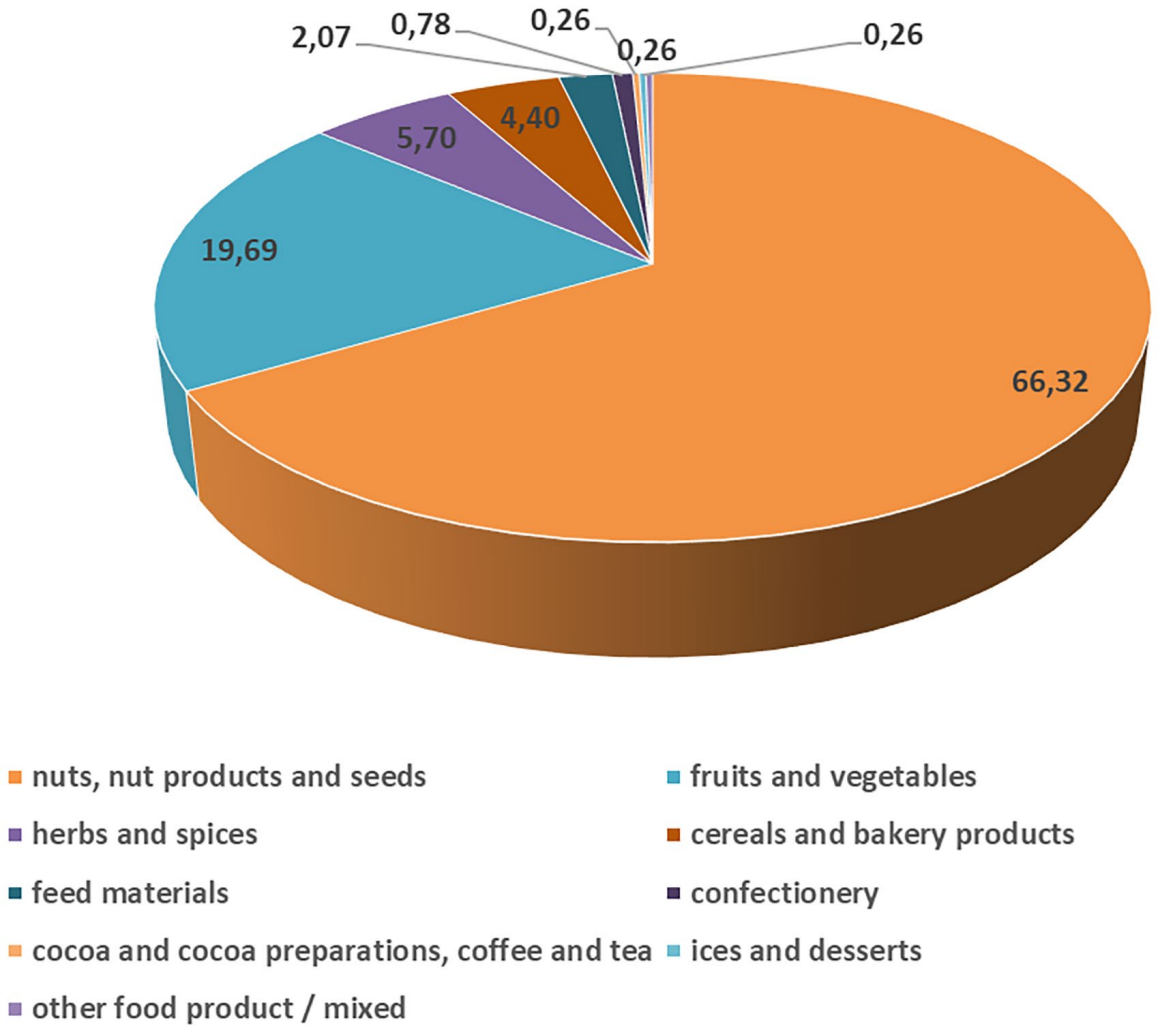
Severe aflatoxin B_1_ contamination for the different food and feed sources (%) reported in the European Union in 2021 (Rapid Alert System for Food and Feed, 2021). The data distribution from the 2021 and 2020 years was not statistically significantly different, but in 2021, the reported cases were twice as much (385) as in 2020 (165). However, the origin of the aflatoxin contaminated products was derived from all over the World. Serious aflatoxin contamination was reported only in some percent every year in the European Union.

Interestingly, AF notifications for the RASFF of the European Union are very low in Europe (Figure 2), especially for maize, compared to the other food sources and other continents, thanks to the regulations. The European Union has one of the most inclusive and sternest regulations on AF levels, set by the commission regulation 1881/2006 (European Commission, 2006) and later by its amending supplement 165/2010 (European Commission, 2010), that are binding upon the 27 member states of the EU. In the current climatic situation, European countries in which maize cultivation is expected, i.e., in France, Romania, North-East Italy and Hungary (in total reporting for 60% of the total production for the 28 EU Member States, FAOStat, 2013), show a low chance of AF occurrence (European Commission, 2007). The European nations trade most of their maize amongst each other; from the outside, several European nations also import maize from Brazil and Argentina and much less from the USA (Wu and Guclu, 2012).

Prediction models are an important tool to predict fungal occurrence and mycotoxin contamination. Nonetheless, there are still open issues, like: (i) keeping prediction accurate in climate change scenario, (ii) remodel the impact of cropping systems, (iii) consider co-occurring fungi and their ecology changing during the growing season, and (iv) multimycotoxin occurrence (Camardo Leggieri et al., 2020).

Various models and datasets related to AFs in maize have been developed and used, but they have not yet been linked. The EU green paper suggested that climate change effects will be regional and be either damaging or beneficial depending on geographical area (European Commission, 2007).

A model reflected a low to a medium probability of AF contamination under the +2°C increase. The climatic changes in Southern Europe are predicted to reach an increase of 4–5°C with more extended drought periods, causing increasing desertification and decreasing crop yields (Solomon et al., 2007). Serious contamination with AF was predicted in some southern European countries like Italy or Bulgaria (Battilani et al., 2016). However, maize production is marginal in these areas. Changes of +2.5–3.5°C with dryer and hotter summers are predicted for Western and Atlantic European areas. For the Central European countries, an increase of 3–4°C, higher rainfall and floods were forecasted, and longer growing periods would benefit crop yields. Northern Europe expects a mean temperature increase of 3–4.5°C and a significant (30–40%) increase in precipitation. It may lead to increases in crop yields and perhaps new crop cultivation techniques and changes in crop successions (European Commission, 2007; Solomon et al., 2007). The +5°C increase was predicted to lead to more expansive contaminated areas in the European domain. However, in southern Europe, AF increase was predicted to be limited since conditions would be less suitable for *A. flavus* growth (Battilani et al., 2016). In 2022, the Intergovernmental Panel on Climate Change reported that the global warming can reach 1.5°C in the near-term (2040) that would cause unavoidable increases in multiple climate hazards and present multiple risks to ecosystems and humans (very high confidence. The frequency of extreme agricultural droughts is projected to be 150 to 200% more likely at 2°C for the Mediterraneum (IPCC, 2022).

## 5. Advanced mycotoxin reduction with biological control

Biological control is regarded as one of the most promising solutions to counteract *Aspergillus* spp. growth in the field. Biological control is achieved by multiple means: (i) parasitism (deriving nutrients from the host); competition (for space and nutrients), and antibiosis (production of inhibitory metabolites and enzymes). The antagonistic behavior may derive from more than one mechanism, depending on the species involved and on the environmental conditions (Calistru et al., 1997). So far many species with *Aspergillus* inhibitory activity have been studied; atoxigenic strains and *Trichoderma* spp. are the most used and effective microorganisms, although bacteria and yeasts showed antagonistic and AFs reducing activities.

### 5.1. Atoxigenic *Aspergillus* strains

Atoxigenic *Aspergillus* strains are able to inhibit toxigenic fungi growth by competitive exclusion and to reduce mycotoxin production thanks to the production of organic volatile compounds (Moore et al., 2021).Atoxigenic *Aspergilli* are not able to produce AFs due to the partial or total deletion in AFs biosynthetic gene cluster (Moore, 2022) and when applied in the field, they can displace toxigenic strains and significantly lower both infection rate and aflatoxin production by native *Aspergilli* (Mauro et al., 2018; Moral et al., 2020). Different commercial products based on single or a combination of atoxigenic strains exist. In the US, *Aspergillus flavus* AF36, developed by USDA-ARS, and Afla-Guard® (*A*.*flavus* NRRL21882), developed by Syngenta, are commercialized. Another success story is Aflasafe® (developed by USDA-ARS, IITA, M&B Gates Foundations et al.), composed by four atoxigenic isolates belonging to distinct vegetative compatibility groups, native to the target nation (Bandyopadhyay et al., 2016). Aflasafe® was authorized in Nigeria, The Gambia, Senegal, Kenia, Burkina Faso, Ghana, Tanzania, and approved in Malawi, Zambia, and Mozambique.

Since 2003, several non-compliant, AF contaminated maize has been produced in Europe due to extreme temperature and dry weather (Mauro et al., 2013; Kos et al., 2018). This prompted the search for atoxigenic strains to be used as biocontrol agents, leading to the development of AF-X1™ (commercialized by Corteva Agriscience), a devitalised sorghum, coated with spores of *A. flavus* MUCL54911 (Mauro et al., 2018). To date, in Italy AF-X1™ has been granted a temporary authorization of 120 days that has to be renewed every year until full authorization as plant protection product from the European commission is granted.

Indeed, despite the high level of efficacy proved in several field trials and the outstanding success in low-income countries (>90% of AFB_1_ reduction), there are still concerns regarding the application of these biocontrol agents in Europe. Data is missing on the viability and population dynamics in water/sediment, its persistence and multiplication in natural environments. Several studies indicate that atoxigenic strains can persist year after year and to reduce aflatoxin contamination even if they are not re-applied (Moore, 2022). The genetic stability of these strains, i.e., if they still are atoxigenic generation after generation, still must be assessed. Additionally, the dietary and non-dietary risk assessments, and the ecotoxicological risk assessment (the impact in terms of pathogenicity and infectivity on non-target organisms, humans included) could not be finalized either due to lack of data (EFSA, 2022).

A concerning issue regards the potential promotion of fumonisin-producing strains and the possibility that non aflatoxigenic strains produce other mycotoxins in the field. Nonetheless, this hypothesis has not been yet verified (Ortega-Beltran et al., 2021).

### 5.2. *Trichoderma* spp.

*Trichoderma* spp. are among the most diffused biocontrol agents worldwide. They are commonly found in soil and root ecosystems, and elicit their biocontrol activities on a wide variety of plant pathogens through mycoparasitism, competition, and antibiosis. In fact, it is a fast growing, metabolically flexible species, able to parasitize other fungi and to produce a wide range of antibiotic substances (Ren et al., 2022).

The active substances produced by *Trichoderma* have shown to reduce *Aspergillus* growth and subsequently AFs production, or possibly to degrade AFs rather than to inhibit their synthesis (Gamal et al., 2022; Ren et al., 2022).

Among the extracellular cell wall degrading enzymes produced by *Trichoderma* spp., peroxidase has been shown to degrade AFs (Dini et al., 2022) and inhibit hyphal growth, while protease P6281 showed inhibitory activity on conidial germination and mycelial growth (Deng et al., 2018).

*Trichoderma* is presently marketed as active ingredients of more than 250 products worldwide, including bio-pesticides, biofertilizers, growth enhancers and stimulants of natural resistance (Woo et al., 2014).

### 5.3. Bacteria and yeasts

Among bacteria, *Bacillus*, *Pseudomonas*, *Lactobacillus*, *Streptomyces* are the main genera with inhibitory activity towards *Aspergillus* spp. mycelial growth, conidial germination, and AFs production by competition and antibiosis. A wide range of inhibitory compounds have been identified among enzymes (chitinases, proteases, and glucanases), peptides, organic acids (lactiv and fatty acids) and miscellaneous alicyclic and aromatic compounds (Ren X. et al., 2020).

Different *Saccharomyces* and non- *Saccharomyces* yeasts (e.g., a *Saccharomyces*, *Aureobasidium*, *Pichia*, *Metschnikowia*, *Dekkera* (van der Walt), and *Rhodotorula* genera) have been studied in biological control, especially for *Aspergillus* infection in grapes (Di Canito et al., 2021). Also yeasts act via competition and antibiosis, the latter via production of lytic enzymes, peptides, toxic compounds, and volatile organic compounds (Ren et al., 2019).

Bacteria and yeast-based commercial biocontrol products are marketed in Europe after approval at EU level and authorization by Member States. Although they have been proven in several papers to be active against *Aspergillus*, they may be specifically registered to be used against other plant pathogens, like *Botrytis cinerea*, depending on the country.

## 6. Advanced decontamination techniques to minimize aflatoxin risk

The growing threat of aflatoxicosis requests novel decontamination measures to ensure food safety and food security. Decontamination techniques can be divided into three categories: (i) chemical, (ii) physical, and (iii) biological, according to the mechanism involved in the reduction or degradation.

Despite the enormous amount of literature published in the last years, there is currently a limited number of valid methods that have been authorized in Europe for aflatoxin reduction. Table 1 summarizes the main characteristics, advantages, and disadvantages of aflatoxin advanced decontamination techniques, which will be further described in the following sections.

**Table 1:** Mode of action, advantages, and disadvantage of aflatoxin decontamination techniques.

Method	Mode of action	Advantages	Disadvantages
Adsorbents	Adsorption	Low level of inclusion; active in the intestinal tract of animals and in feed; functionalization allows the binding towards different toxins.	Unspecific binding of nutrients, medicinal drugs, vitamins *etc*.
Microorganisms	Adsorption and biotransformation	Work through two modes of action; Multistep processes may be performed; multimycotoxin activity is possible; Inexpensive.	Impact on food matrix may be relevant; production of multiple (undesired) metabolites; not applicable to all matrices.
Enzymes	Biotransformation	Minor impact on food matrix; Specific; multistep processes may not be performed by single enzymes; Multimycotoxin activity is unlikely.	Expensive; formulation is needed to ensure activity in harsh environments/in vivo; lack of knowledge on residual toxicity of degradation products.
Ultraviolet irradiation	Biotransformation	Effective using mild operational conditions, low cost, high operability at industrial scale; already in use for surface sanitization in food industry.	low penetration in solids and in turbid liquids; lack of knowledge on residual toxicity of degradation products.
Magnetic nanoparticles	Adsorption and biotransformation	Work through two modes of action; easy recovery; can be designed/functionalized for multimycotoxin binding activity.	lack of knowledge on the effects on food/feed matrices; residual toxicity of degradation products and of magnetic particles.
Plasma treatment	Biotransformation	Also inactivates fungal growth and mycotoxin production.	High cost of equipment; low penetration in solids and in turbid liquids; lack of knowledge on the effects on food/feed matrices; lack of knowledge on residual toxicity of degradation products.
Nanozymes	Adsorption and biotransformation	Work through two modes of action; high efficiency, stability, and reusability	lack of knowledge on the effects on food/feed matrices; lack of knowledge on residual toxicity of degradation products.

### 6.1. Present-day methods: Mycotoxin detoxifying agents

Mycotoxin detoxifying agents (MDAs) were defined by the EFSA (European Food Safety Authority) et al. (2022) as “substances that can suppress or reduce the absorption, promote the excretion of mycotoxins or modify their mode of action” (Boudergue et al., 2019). Therefore, two main categories were identified, namely adsorbing and biotransforming agents. Adsorbing agents reduce mycotoxin bioavailability and uptake in living organisms by physically binding to the toxin, while biotransforming agents degrade mycotoxins into non-toxic metabolites (Boudergue et al., 2019).

From a regulatory point of view, Commission Regulation (EC 1831/2003; European Commission, 2003) introduced the category of technical additives intended to “suppress or reduce the absorption, promote the excretion of mycotoxins or modify their mode of action.” Nonetheless, detoxification processes can be applied in Europe to products intended for animal feed (Commission Regulation 786/2015; European Commission, 2015) if it is effective, characterized, does not result in harmful residues of both the substance/microorganism/enzyme used to detoxify and the toxin, and does not adversely affect the characteristics and the nature of the feed. This implies that a safety and efficacy assessment must be carried out before a method can be proposed and commercialized. Aside from these requirements, the cost of such a method must be considered, especially when treating a low-cost commodity, like, for example, maize for feed.

#### 6.1.1. Adsorbents

Adsorbents may be silica-based inorganic compounds or carbon-based organic polymers and can be used to sequester mycotoxins in feed or the gut of feedstock. Commercially available inorganic adsorbents are composed of aluminosilicates, such as bentonite, montmorillonite, zeolite, and hydrated sodium calcium aluminosilicates (HSCAS), and they can bind the β-keto-lactone or bilactone system of AFs through the uncoordinated metal ions in the mineral (Čolović et al., 2019). Carbon-based organic polymers include yeast cell wall and glucomannan, composed of lipids, proteins, polysaccharides, glucans and mannans. Being chemically and physically diverse compounds, they bind AFB_1_ through different types of non-covalent interactions, such as hydrogen bonds, and ionic or hydrophobic interactions (Kolawole et al., 2019).

The adsorption capability depends on the charge distribution, surface area, and pore size of the material. It may also occur within the layers of the adsorbent, thus increasing its binding capacity (Zhu et al., 2016). Adsorbents are widely commercialized as technical additives and proven to reduce the detrimental effects of AFs ingestion.

Despite being incredibly effective in binding AFB_1_, adsorbents display several disadvantages, like the unspecific binding to micro and macronutrients, veterinary medicinal products and additives, and the narrow spectrum of action towards multiple mycotoxins. To overcome this issue, research focuses on developing innovative hybrid adsorbents which combine the physical properties and binding capacity of different materials (Ma, 2019). Nano-adsorbents are a hot research topic because they have improved selectivity, sensitivity, and binding area. Graphene derivatives and clay minerals are the most promising materials because of their efficacy and lower cost. Nonetheless, large-scale production and commercialization are limited due to scale-up difficulties, high cost, and lack of long-term toxicity studies (Song and Qin, 2022).

Several recent papers investigated the potential of probiotics in AFs binding and, to a less extent, degradation, with regards to *Lactobacillus* spp., *Lactocaseibacillus* spp., *Streptococcus* spp., and *Saccharomyces cerevisiae*. The peptidoglycans and other cell wall polysaccharides are responsible of binding AFs. They show great potential even for a possible food application, considering their Qualified Presumption of Safety (QPS) status (Abdolmaleki et al., 2022).

#### 6.1.2. Microorganisms

Microbial detoxification of mycotoxins has been known since a very long time. The evidence that mycotoxins do not accumulate in agricultural soil and that can be reduced in some fermented foods prompted very productive research on microorganisms able to degrade mycotoxins and, consequently, on enzymes able to perform such degradation (Zhu et al., 2017). The isolation of microorganisms is often performed from contaminated environments, and followed by an enrichment step, in which the microorganism is grown in specific selective media which contain restricted carbon sources and a high level of mycotoxin contamination.

Microorganisms can reduce mycotoxin contamination by two different mechanisms, namely adsorption to cell wall, as discussed in Section “Atoxigenic Aspergillus strains,” and biotransformation. Biotransformation of AFs can be performed by different genera of filamentous fungi (*Pleurotus*, *Armillariella*, *Armoracia*, *Trametes*, *Rhizopus*, *Trichoderma*, *Clonostachys*, *Cladosporium*, *Aspergillus*), yeasts (*Saccharomyces*, *Pichia*, *Candida*, *Kluyveromyces*, *Yarrowia*, *Rhodotorula*, *Rhodosporidium*), and bacteria (*Bacillus*, *Metschnikowia*, *Komagataella*, *Streptomyces*, *Rhodococcus*, *Pseudomonas*, *Pediococcus*, *Lactiplantibacillus*, *Lactiplantibacillus*, *Enterobacter*, *Cupriavidus*, *Brevibacterium*) (Piotrowska, 2021; Nahle et al., 2022).

Currently, two commercial products based on living microorganisms have been released onto the market. A novel genus, formerly known as *Eubacterium*, belonging to the *Coriobacteriaceae* family (Biomin® BBSH® 797, DSM 11798) produces de-epoxidases able to detoxify trichothecenes, and *Trichosporon mycotoxinivorans*, a non-pathogenic yeast, produces peptidases able to detoxify ochratoxin A. However, so far, there is not any valid, commercially available microorganism-based solution to decontaminate AFs in feed.

Using whole microbial cells instead of the isolated enzyme has the advantage of being low-cost and potentially more effective. A single organism can produce enzymes able to degrade different toxins, or to perform multi step reactions. Indeed, as described by Heinl et al. (2010), fumonisin degradation can be performed by the consecutive action of a carboxylesterase and an aminotransferase. On the other hand, the production of degrading enzymes by microorganism may be difficult to standardize and to adjust to the level of contamination. Other enzymes and metabolites can be produced and their effect on the properties of the matrix might be difficult to predict. The only exception is represented by matrices specifically intended to be fermented, like silage, or by-products which can be fermented and used as ingredients in feeds, like spent grains or distilled dried grains with solubles (DGGS). For these reasons, the use of isolated enzymes represents a more convenient, though expensive option.

#### 6.1.3. Enzymes

A wide variety of enzymes have been identified and characterized for their mycotoxin degrading capabilities in the last years (Loi et al., 2017; Wang et al., 2022).

So far only two enzyme-based commercial products have been released onto the European market. Fumzyme® by DSM is a fumonisin esterase capable of degrading fumonisin B_1_ to a non-toxic compound, which has been recently approved for use in all animal species (EFSA Panel on Additives, and Products or Substances used in Animal Feed (FEEDAP) et al., 2020; Regulation EU, 2021/363). ZENzyme® by DSM is a hydrolase able to detoxify zearalenone, recently approved for all terrestrial animal species (EFSA Panel on Additives, and Products or Substances used in Animal Feed (FEEDAP) et al., 2022).

There are no other successful examples, especially for AFs. Despite the many publications on AFs degradation by enzymes (Loi et al., 2016, 2020a; Yang et al., 2021), currently there are no commercial options onto the market. Most of the literature focuses on *in vitro* AFs degradation, lacks data on degradation products, and toxicity assessments.

Most AFs degrading enzymes reported in literature fall into the category of oxidoreductases, like laccases, peroxidase, or the so-called “AF oxidases” (Loi et al., 2017; Guan et al., 2021; Kumar et al., 2022). AF’s structure is highly stable and requires a strong oxidation to be degraded. These enzymes are incredibly versatile, yet unspecific. This complicates to study the mechanism, kinetics, degradation products, and toxicity of the compounds resulting from the reaction. AFs are metabolized in *in vivo* by endogenous oxidases, which activate AFB_1_ to the mutagenic 8,9-epoxyAFB_1_. This is performed by specific liver cytochromes P450 isoforms (CYP1A2, 2A6, and 2A13). Nonetheless, unspecific oxidation might also result in the generation of AFQ_1_, a less toxic compound, with other specific cytochrome isoforms (CYP3A4, 1A2, 3A7). Interestingly, it has been demonstrated that peroxidases and laccase-like enzymes can detoxify AFB_1_ to AFQ_1_ and do not activate the toxin to epoxide (Loi et al., 2020b; Qin et al., 2021). Other degradation mechanisms have been hypothesized, including the nucleophilic attack on the lactone and the furan rings, leading to their opening (Kumar et al., 2022). Although the enzymatic reduction has always been referred to be specific, this is not true for these enzymes. Their capability of degrading AFs relies on their high oxidative capacity, which is not restricted to AFs as substrates. Laccases and peroxidases also work in combination with redox mediators, which broaden even more the substrate spectrum of these enzymes. Indeed, when studied in protein and carbohydrate-rich foods, these enzymes have been shown to catalyze the formation of crosslinks and deeply modify the nutritional, technological, and rheological properties of foods (Isaschar-Ovdat and Fishman, 2018). In some cases, the modification resulted in an improvement of the nutritional (Loi et al., 2018) and technological properties (Loi et al., 2020a,b) of the food, therefore a careful case by case evaluation is necessary to effectively apply these enzymes.

Stability and activity in real matrices are challenges for the enzymes, and structure-based engineering has been shown to dramatically improve enzyme performance (Wang et al., 2022). The cost of enzyme production is another important hustle to overcome. Recombinant production is an effective strategy to increase production yield and lower costs.

### 6.2. Innovative methods

#### 6.2.1. Ultraviolet irradiation

The use of ultraviolet radiation (UV) proved to be an effective physical method to reduce contaminants and microorganisms through photochemical degradation and DNA damage, respectively (Sun et al., 2019). Ultraviolet radiation (UV) is a non-ionized radiation, with wavelength ranges between 100 and 400 nm. UV light spectrum is divided UV-A (315–400 nm), UV-B (280–315 nm), UV-C (200–280 nm), and UV-V (100–200 nm) (Rifna et al., 2019) and may be generated by solar radiation, UV lamps, or even Light Emitting Diodes (LEDs). LEDs show unique advantages, especially regarding the lack of radiant heat and can be easily applied in refrigerated storage (Loi et al., 2021). Due to its antibacterial properties, it finds application in the food industry to reduce the microbial load in air and water or on the surface of fresh products like fish, egg, chicken, liquid food, milk, fruit juices, or cider (Akhila et al., 2021). Two different mechanisms have been described in UV irradiation mediated degradation of contaminants: photolysis and photocatalysis. In the first case, degradation is due to the absorption of electromagnetic radiation, while in the latter, a photocatalyst (metal oxide, metal chalcogenide, or carbon-based material) is used to enhance degradation: TiO_2_ and ZnO are among the most used catalysts.

Photolysis occurs when reactant molecules absorb photons; electrons are excited to a high energy state, and when energy is released the chemical structure of the molecule is altered. The wavelength of UV radiation is inversely proportional to photon energy. Therefore, UV-C radiation has shown the most promising effects for microbial and mycotoxins decontamination (Shen and Singh, 2021).

UV light can also be given in a concentrated form, so that intense short bursts (pulses) have more penetration capacity. This technology is therefore called “pulsed UV.”

Aflatoxins are photosensitive and may be degraded by either photolysis or photocatalysis. AFB_1_ photocatalytic degradation is supposed to occur by direct oxidization of hydroxyl free radicals (•OH), H^+^, and other oxidative species generated by UV/photocatalyst. UV treatment leads to profound modifications in the chemical structure of AFB_1_, which preferentially start from the double bond on the bifuran moiety. Other degradation pathways involve the cycloaddition reaction at the furan ring and lactone ring, followed by further structure rearrangements (Sun et al., 2019; Murugesan et al., 2021; Song et al., 2022).

UV treatment has been employed for mycotoxins degradation in food commodities, such as oils, milk, wheat, and peanut. UV photocatalysis showed several limitations when applied to food, such as the oxidation of valuable nutritional components and low penetration in solids and turbid liquids (Shen and Singh, 2022). UV photolysis/photocatalysis is considered as an advanced oxidation method with significant advantages: no secondary pollution, easy and mild operational conditions, low cost, operability, and applicability in the food industry (Shen and Singh, 2022). For these reasons, it has been employed with success for the degradation of organic pollutants in the wastewater and air environment. Nonetheless, due to its oxidative nature, a careful evaluation of the detrimental effect on food in terms of sensory and nutritional profiles and toxin residues must be performed to achieve a satisfactory application. Scale-up of the technology for bulk-level applications, sustainability, and reusability of photocatalysts are important features that deserve further study (Magzoub et al., 2019).

#### 6.2.2. Magnetic nanoparticles

Magnetic nanoparticles are small particles (less than 100 nm) made up of pure metals, metal alloys and metal oxides which have emerged as excellent adsorbents, due to their unique structural advantages, large surface area, tunable surface functionalities, and easy recovery with external magnetic fields (Horky et al., 2018).

Iron and zinc oxides, silver, copper, or selenium nanoparticles are gaining massive attention in mycotoxin research because of their effective binding capacity in agricultural feedstuff and foods. Nanoparticles can be functionalized to enhance mycotoxin binding capacity, to provide binding affinity towards various types of mycotoxins, or even to immobilize enzymes, cells (Duishemambet Kyzy et al., 2022) or build magnetic-propelled yeast cell robots (Lu et al., 2021) able to reduce mycotoxin contamination. AFB_1_ degradation has been studied using iron oxide nanoparticles *in vitro* and in edible oils magnetic graphene composite. The main issue for the practical use such material is the gap in the toxicity evaluation and in data collection to set safety limits (Malhotra et al., 2020).

#### 6.2.3. Plasma treatment

Plasma is an ionized gas that generates several reactive charged and neutral species, including photons, positive and negative ions, and oxygen and nitrogen reactive species (Mandal et al., 2018). It can be divided into thermal and non-thermal (cold) plasma, depending on the type of gas generation methods, and working temperature. Cold plasma works at around room temperature (30–60°C), and for this reason, it finds multiple applications in sterilization, inactivation, decontamination, and disinfection in the food industry. The reactive species generated by the cold plasma are highly active oxidants.

The capability of cold plasma to inactivate fungal growth and mycotoxin production has been well documented. Nonetheless, recently some studies have also investigated the capability of degrading mycotoxins (Hojnik et al., 2021; Wu et al., 2021). The degradation mechanism has been recently unraveled: oxidative degradation of AFB_1_ occurred via the electrophilic addition of water on C8 and formation of AFB_2a_ or H-atom abstraction from the bonds C–H at the 8 and 9 positions. These reactions lead to the opening of the lactone and terminal furan ring; these unstable intermediates undergo further degradation (Hojnik et al., 2021; Li et al., 2022).

Plasma technology is at its very first beginning, and there are substantial limitations to its concrete application. Suitable plasma equipment is still at the laboratory stage and mainly designed for other applications besides food. The process still needs standardization and improvement to overcome the low penetration capacity (Wu et al., 2021). Finally, the effects on food matrices have been poorly studied, and further research is needed to propose this technology as an AF decontamination method in food and feed.

#### 6.2.4. Nanozymes

Nanozymes are inorganic nanoparticles with enzyme-like properties in redox reactions. They combine the properties of nanomaterials and oxidases in a more stable and efficient system. Nanozymes with laccase and peroxidase-like activities were developed for contaminant removal, including AFB_1_ (Zhang et al., 2020; Guo et al., 2021; Wei et al., 2022). Nanozyme show also adsorptive characteristics due to the hierarchical porous structure (Wu et al., 2020; Ma et al., 2021; Pérez-Gómez et al., 2022) and can combine filtration, adsorption, and catalysis in a multifunctional removal process (Ren et al., 2019). These studies show that these innovative materials possess high efficiency (up to 96%), stability, and reusability (up to 5 cycles). Few authors investigated the effectiveness on real matrices (Ma et al., 2021; Wei et al., 2022) and the toxicity of both nanozyme and degradation products (Wei et al., 2022). These data are promising, as high removal rates were obtained in vegetable oils, with little impact on their quality. In the study conducted by Wei et al. (2022), the stability of the metal part was assessed to verify that no metal component leaked into the food.

Even though preliminary LC–MS/MS data suggest that less toxic compounds could be generated by nanozyme catalysis, additional research is needed to further characterize these products and confirm that they are less toxic. On the other hand, there is no data on their use in solid, low-water content materials like grains and nuts, which would represent the most important and useful application for AFs removal.

### 6.3. Gaps and main limitations of postharvest decontamination techniques

Despite the technical obstacles, there are several steps which must be considered for a successful development of a new post-harvest reduction method.

A mandatory prerequisite to research for a new product and invest in its commercial application is that there is a sufficient need, an end-user, and an economic benefit for both the producer and the final customer. Even if aflatoxin threat is *per se* a sufficient reason to motivate the research and development of new post-harvest reduction methods, the low perceived risk of incurring aflatoxicosis, the low cost of the raw material and the possibility to downgrade or divert the material to countries with less stringent regulation, might slow the process of developing new postharvest methods. A close partnership between research and companies is mandatory to overcome technical and economical gaps between research and practical application.

So far, the cost-effectiveness and technology readiness level (TRL) of innovative postharvest methods are weak points (Marshall et al., 2020). Some of the innovative technologies discussed in this review might be already in use and commercially available for different applications; nonetheless, they have low TRLs (3–5) if we consider mycotoxin reduction application (Marshall et al., 2020).

Large-scale production and the impact of such methods in actual application must be assessed (Ortega-Beltran and Bandyopadhyay, 2021). Another critical point is that regulatory approval through a dossier submission to EFSA must be obtained. Nonetheless, the biggest obstacle is still represented by the regulatory gap that hinders the application of such methods to food matrices.

## 7. Conclusion

AFs contamination is becoming an emerging risk in European countries and requires the implementation of novel post-harvest methods. Due to climate change and growing populations, environmental factors affecting mycotoxins production are also altering. This leads to a change in the distribution and growth of fungi in the soil which is difficult to predict and prevent.

AFs are among the most difficult toxin to degrade, and effective degradation only occurs when strong oxidants are used, irrespectively of their nature (physical or biological methods) and origin. Adsorption, on the other hand, can be used for AFs removal and commercial solutions exist for feed use. The toxicological impact and detrimental effects on food matrices still need to be deeply investigated for new technologies.

Adopting an integrated pre- and post-harvest approach, possibly using different reduction techniques remains the most effective way to counteract AF risk. None of the strategies can completely prevent or reduce AFs contamination or can be used as a general all-purpose decontamination method. Future research is needed to overcome gaps and limitations, possibly in close connection with industries and other stakeholders, to finally apply these novel methods at the industrial level.

## Author contributions

AL and IP contributed to the conception and design of the paper. All authors wrote the first draft and contributed to the manuscript revision, read, and approved the submitted version.

## Funding

This work was supported by Project no. 2018-1.2.1-NKP-2018-00002 which has been implemented with the support provided from the National Research, Development and Innovation Fund of Hungary, financed under the 2018-1.2.1-NKP funding scheme, by Project no. TKP2021-EGA-20 810 (Biotechnology), which has been implemented with the support provided from the National Research, Development and Innovation Fund of Hungary, financed under the TKP2021-EGA funding scheme, by the National Research, Development and Innovation Office of Hungary with the grant K142801. This project has received funding from the European Union’s Horizon 2020 research and innovation programme under grant agreement No 952337.

## Conflict of interest

The authors declare that the research was conducted in the absence of any commercial or financial relationships that could be construed as a potential conflict of interest.

## Publisher’s note

All claims expressed in this article are solely those of the authors and do not necessarily represent those of their affiliated organizations, or those of the publisher, the editors and the reviewers. Any product that may be evaluated in this article, or claim that may be made by its manufacturer, is not guaranteed or endorsed by the publisher.

## References

[ref1] Abbas H. K. Accinelli C. Shier W. T. (2017). Biological control of aflatoxin contamination in US crops and the use of bioplastic formulations of *Aspergillus flavus* biocontrol strains to optimize application strategies. J. Agric. Food Chem. 65, 7081–7087. doi: 10.1021/acs.jafc.7b01452 28420231

[ref2] Abdel-Hadi A. M. Caley D. P. Carter D. R. F. Magan N. (2011). Control of aflatoxin production of *Aspergillus flavus* and *Aspergillus parasiticus* using RNA silencing technology by targeting *aflD* (nor-1) gene. Toxins 3, 647–659. doi: 10.3390/toxins3060647, PMID: 22069731 PMC3202845

[ref3] Abdel-Hadi A. Carter D. Magan N. (2010). Temporal monitoring of the nor-1 (*aflD*) gene of *Aspergillus flavus* in relation to aflatoxin B_1_ production during storage of peanuts under different water activity levels. J. Appl. Microbiol. 109, 1914–1922. doi: 10.1111/j.1365-2672.2010.04820.x, PMID: 20735510

[ref4] Abdel-Hadi A. Schmidt-Heydt M. Parra R. Geisen R. Magan N. (2012). A systems approach to model the relationship between aflatoxin gene cluster expression, environmental factors, growth and toxin production by *Aspergillus flavus*. J. R. Soc. Interface 9, 757–767. doi: 10.1098/rsif.2011.0482, PMID: 21880616 PMC3284141

[ref5] Abdolmaleki K. Javanmardi F. Gavahian M. Phimolsiripol Y. Ruksiriwanich W. Mir S. A.. (2022). Emerging technologies in combination with probiotics for aflatoxins removal: an updated review. Int. J. Food Sci. 57, 5712–5721. doi: 10.1111/ijfs.15926

[ref6] Adhikari B. N. Bandyopadhyay R. Cotty P. J. (2016). Degeneration of aflatoxin gene clusters in *Aspergillus flavus* from Africa and North America. AMB Express 6, 62–16. doi: 10.1186/s13568-016-0228-6, PMID: 27576895 PMC5005231

[ref7] Agbetiameh D. Ortega-Beltran A. Awuah R. T. Atehnkeng J. Islam M. S. Callicott K. A.. (2019). Potential of atoxigenic *Aspergillus flavus* vegetative compatibility groups associated with maize and groundnut in Ghana as biocontrol agents for aflatoxin management. Front. Microbiol. 10:2069. doi: 10.3389/fmicb.2019.02069, PMID: 31555251 PMC6743268

[ref010] Akhila P. P. Sunooj K. V. Aaliya B. Navaf M. Sudheesh C. Sabu S.. (2021). Application of electromagnetic radiations for decontamination of fungi and mycotoxins in food products: A comprehensive review. Trends in Food Science & Technology 114, 399–409. doi: 10.1016/j.tifs.2021.06.013

[ref8] Amaike S. Keller N. P. (2011). Aspergillus flavus. Annu. Rev. Phytopathol. 49, 107–133. doi: 10.1146/annurev-phyto-072910-095221 21513456

[ref9] Amare M. G. Keller N. P. (2014). Molecular mechanisms of *Aspergillus flavus* secondary metabolism and development. Fungal Genet. Biol. 66, 11–18. doi: 10.1016/j.fgb.2014.02.008, PMID: 24613992

[ref10] Apel K. Hirt H. (2004). Reactive oxygen species: metabolism, oxidative stress, and signal transduction. Annu. Rev. Plant Biol. 55, 373–399. doi: 10.1146/annurev.arplant.55.031903.141701 15377225

[ref11] Arias R. S. Dang P. M. Sobolev V. S. (2015). RNAi-mediated control of aflatoxins in peanut: method to analyze mycotoxin production and transgene expression in the peanut/*Aspergillus* pathosystem. JoVE 106:e53398. doi: 10.3791/53398, PMID: 26709851 PMC4694054

[ref013] Bai Y. Wang S. Zhong H. Yang Q. Zhang F. Zhuang Z.. (2015). Integrative analyses reveal transcriptome-proteome correlation in biological pathways and secondary metabolism clusters in *A. flavus* in response to temperature. Sci. Rep. 5:14582. 26416011 10.1038/srep14582 PMC4586720

[ref12] Baidya S. Duran R. M. Lohmar J. M. Harris-Coward P. Y. Cary J. W. Hong S. Y.. (2014). VeA is associated with the response to oxidative stress in the aflatoxin producer *Aspergillus flavus*. Eukaryot. Cell 13, 1095–1103. doi: 10.1128/EC.00099-14, PMID: 24951443 PMC4135802

[ref13] Bandyopadhyay R. Ortega-Beltran A. Akane A. Mutegi C. Atehnkeng J. Kaptoge L.. (2016). Biological control of aflatoxins in Africa: current status and potential challenges in the face of climate change. World Mycotoxin J. 9, 771–789. doi: 10.3920/WMJ2016.2130

[ref14] Battilani P. Toscano P. der Fels-Klerx V. Moretti A. Camardo Leggieri M. Brera C.. (2016). Aflatoxin B_1_ contamination in maize in Europe increases due to climate change. Sci. Rep. 6, 1–7. doi: 10.1038/srep24328(2016) 27066906 PMC4828719

[ref15] Bayram Ö. Braus G. H. (2012). Coordination of secondary metabolism and development in fungi: the velvet family of regulatory proteins. FEMS Microbiol. Rev. 36, 1–24. doi: 10.1111/j.1574-6976.2011.00285.x, PMID: 21658084

[ref16] Boudergue C. Burel C. Dragacci S. Favrot M. C. Fremy J. M. Massimi C.. (2019). Review of mycotoxin-detoxifying agents used as feed additives: mode of action, efficacy and feed/food safety. EFSA Support. Publ. 6:22E. doi: 10.2903/sp.efsa.2009.EN-22

[ref17] Buchanan R. L. Ayres J. C. (1977). Effect of various glycolytic and TCA intermediates on aflatoxin production. J. Food Saf. 1, 19–28. doi: 10.1111/j.1745-4565.1977.tb00256.x

[ref18] Caceres I. El Khoury R. Bailly S. Oswald I. P. Puel O. Bailly J. D. (2017). Piperine inhibits aflatoxin B_1_ production in *Aspergillus flavus* by modulating fungal oxidative stress response. Fungal Genet. Biol. 107, 77–85. doi: 10.1016/j.fgb.2017.08.005, PMID: 28830793

[ref19] Caceres I. Khoury A. A. Khoury R. E. Lorber S. Oswald I. P. Khoury A. E.. (2020). Aflatoxin biosynthesis and genetic regulation: a review. Toxins 12:150. doi: 10.3390/toxins12030150, PMID: 32121226 PMC7150809

[ref20] Calistru C. McLean M. Berjak P. (1997). *In vitro* studies on the potential for biological control of *Aspergillus flavus* and *Fusarium moniliforme* by *Trichoderma* species. Mycopathologia 137, 115–124. doi: 10.1023/A:1006802423729, PMID: 16284721

[ref012] Calvo A. M. (2008). The VeA regulatory system and its role in morphological and chemical development in fungi. Fungal Genet. Biol. 45, 1053–1061. 18457967 10.1016/j.fgb.2008.03.014

[ref21] Calvo A. M. Bok J. W. Brooks W. Keller N. P. (2004). Vea is required for toxin and sclerotial production in *Aspergillus parasiticus*. Appl. Environ. Microbiol. 70, 4733–4739. doi: 10.1128/AEM.70.8.4733-4739.2004, PMID: 15294809 PMC492383

[ref22] Calvo A. M. Wilson R. A. Bok J. W. Keller N. P. (2002). Relationship between secondary metabolism and fungal development. MMBR 66, 447–459. doi: 10.1128/MMBR.66.3.447-459.2002 12208999 PMC120793

[ref23] Camardo Leggieri M. Lanubile A. DallAsta C. Pietri A. Battilani P. (2020). The impact of seasonal weather variation on mycotoxins: maize crop in 2014 in northern Italy as a case study. World Mycotoxin J. 13, 25–36. doi: 10.3920/WMJ2019.2475

[ref24] Cary J. W. Entwistle S. Satterlee T. Mack B. M. Gilbert M. K. Chang P. K.. (2019). The transcriptional regulator Hbx1 affects the expression of thousands of genes in the aflatoxin-producing fungus *Aspergillus flavus*. G3: Genes Genom. Genet. 9, 167–178. doi: 10.1534/g3.118.200870.Doi:10.1534/g3.118.200870 PMC6325891 30425054

[ref25] Cary J. W. Harris-Coward P. Y. Ehrlich K. C. Mack B. M. Kale S. P. Larey C.. (2012). NsdC and NsdD affect *Aspergillus flavus* morphogenesis and aflatoxin production. Eukaryot. Cell 11, 1104–1111. doi: 10.1128/EC.00069-12 22798394 PMC3445981

[ref26] Chanda A. Roze L. V. Linz J. E. (2010). A possible role for exocytosis in aflatoxin export in *Aspergillus parasiticus*. Eukaryot. Cell 9:1727. doi: 10.1128/EC.00118-10 PMC2976301 20870882

[ref27] Chang P. K. (2022). *Aspergillus flavus* La3279, a component strain of the Aflasafe™ biocontrol product, contains a partial aflatoxin biosynthesis gene cluster followed by a genomic region highly variable among *A. flavus* isolates. Int. J. Food Microbiol. 366:109559. doi: 10.1016/j.ijfoodmicro.2022.109559 35144216

[ref28] Chang P. K. Abbas H. K. Weaver M. A. Ehrlich K. C. Scharfenstein L. L. Cotty P. J. (2012a). Identification of genetic defects in the atoxigenic biocontrol strain *Aspergillus flavus* K49 reveals the presence of a competitive recombinant group in field populations. Int. J. Food Microbiol. 154, 192–196. doi: 10.1016/j.ijfoodmicro.2012.01.005 22285533

[ref29] Chang P.-K. Ehrlich K. C. Yu J. Bhatnagar D. Cleveland T. E. (1995). Increased expression of *Aspergillus parasiticus aflR*, encoding a sequence- specific DNA-binding protein, relieves nitrate inhibition of aflatoxin biosynthesis. Appl. Environ. Microbiol. 61, 2372–2377. doi: 10.1128/aem.61.6.2372-2377.1995 7793958 PMC167509

[ref30] Chang P. K. Scharfenstein L. L. Ehrlich K. C. Wei Q. Bhatnagar D. Ingber B. F. (2012b). Effects of *laeA* deletion on *Aspergillus flavus* conidial development and hydrophobicity may contribute to loss of aflatoxin production. Fungal Biol. 116, 298–307. doi: 10.1016/j.funbio.2011.12.003 22289775

[ref31] Chang P. K. Yu J. Yu J. H. (2004). *aflT*, a MFS transporter-encoding gene located in the aflatoxin gene cluster, does not have a significant role in aflatoxin secretion. Fungal Genet. Biol. 41, 911–920. doi: 10.1016/j.fgb.2004.06.007 15341913

[ref32] Čolović R. Puvača N. Cheli F. Avantaggiato G. Greco D. Đuragić O.. (2019). Decontamination of mycotoxin-contaminated feedstuffs and compound feed. Toxins 11:617. doi: 10.3390/toxins11110617 31731462 PMC6891401

[ref33] Cruz de Carvalho M. H. (2008). Drought stress and reactive oxygen species: production, scavenging and signaling. Plant Signal. Behav. 3, 156–165. doi: 10.4161/psb.3.3.5536 19513210 PMC2634109

[ref03] Deng J. J. Huang W. Q. Li Z. W. Lu D. L. Zhang Y. Luo X. C. (2018). Biocontrol activity of recombinant aspartic protease from *Trichoderma harzianum* against pathogenic fungi. Enzyme and microbial technology 112, 35–42. doi: 10.1016/j.enzmictec.2018.02.002 29499778

[ref34] Di Canito A. Mateo-Vargas M. A. Mazzieri M. Cantoral J. Foschino R. Cordero-Bueso G.. (2021). The role of yeasts as biocontrol agents for pathogenic fungi on postharvest grapes: a review. Foods 10:1650. doi: 10.3390/foods10071650 34359520 PMC8306029

[ref35] Dini I. Alborino V. Lanzuise S. Lombardi N. Marra R. Balestrieri A.. (2022). *Trichoderma* enzymes for degradation of aflatoxin B_1_ and ochratoxin A. Molecules 27:3959. doi: 10.3390/molecules27123959 35745082 PMC9231114

[ref36] Dövényi-Nagy T. Rácz C. Molnár K. Bakó K. Szláma Z. Jóźwiak Á.. (2020). Pre-harvest modelling and mitigation of aflatoxins in maize in a changing climatic environment—a review. Toxins 12:768. doi: 10.3390/toxins12120768 33291729 PMC7761929

[ref37] Drott M. T. Satterlee T. R. Skerker J. M. Pfannenstiel B. T. Glass N. L. Keller N. P.. (2020). The frequency of sex: population genomics reveals differences in recombination and population structure of the aflatoxin-producing fungus *Aspergillus flavus*. MBio 11, e00963–e00920. doi: 10.1128/mBio.00963-20 32665272 PMC7360929

[ref38] Duishemambet Kyzy A. Kocyigit Y. Ardag A. H. (2022). Aflatoxin B_1_ bioremoval by fungal cells immobilised on magnetic nanoparticles. J. Environ. Anal. Chem., 1–14. doi: 10.1080/03067319.2022.2115897

[ref39] Duran R. M. Cary J. W. Calvo A. M. (2007). Production of cyclopiazonic acid, aflatrem, and aflatoxin by *Aspergillus flavus* is regulated by *veA*, a gene necessary for sclerotial formation. Appl. Microbiol. Biotechnol. 73, 1158–1168. doi: 10.1007/s00253-006-0581-5 16988822

[ref40] Dutton M. F. (1988). Enzymes and aflatoxin biosynthesis. Microbiol. Rev. 52, 274–295. doi: 10.1128/mr.52.2.274-295.1988 3137428 PMC373139

[ref41] EFSA (European Food Safety Authority) Alvarez F. Arena M. Auteri D. Binaglia M. C. A. Chiusolo A.. (2022). Conclusion on the peer review of the pesticide risk assessment of the active substance *Aspergillus flavus* strain MUCL54911. EFSA J. 20:7202:21. doi: 10.2903/j.efsa.2022.7202 PMC8968767 35386571

[ref42] EFSA Panel on Additives, and Products or Substances used in Animal Feed (FEEDAP) Bampidis V. Azimonti G. Bastos M. D. L. Christensen H. Dusemund B.. (2020). Safety and efficacy of fumonisin esterase from *Komagataella phaffii* DSM 32159 as a feed additive for all animal species. EFSA J. 18:e06207. doi: 10.2903/j.efsa.2018.5269 32699560 PMC7369623

[ref43] EFSA Panel on Additives, Products or Substances used in Animal Feed (FEEDAP) Bampidis V. Azimonti G. MDL B. Christensen H. Dusemund B.. (2022). Safety and efficacy of a feed additive consisting of zearalenone hydrolase produced by *Escherichia coli* DSM 32731 for all terrestrial animal species (Biomin GmbH). EFSA J. 20:e07157. doi: 10.2903/j.efsa.2022.7157 35233253 PMC8867527

[ref44] Ehrlich K. C. Cary J. W. Montalbano B. G. (1999). Characterization of the promoter for the gene encoding the aflatoxin biosynthetic pathway regulatory protein AflR. Biochim. Biophys. Acta Gene Struct. Expr. 1444, 412–417. doi: 10.1016/s0167-4781(99)00022-6 10095064

[ref45] Einolghozati M. Talebi-Ghane E. Ranjbar A. Mehri F. (2021). Concentration of aflatoxins in edible vegetable oils: a systematic meta-analysis review. Eur. Food Res. Technol. 247, 2887–2897. doi: 10.1007/s00217-021-03844-5

[ref46] Eom T. J. Moon H. Yu J. H. Park H. S. (2018). Characterization of the velvet regulators in *Aspergillus flavus*. J. Microbiol. 56, 893–901. doi: 10.1007/s12275-018-8417-4, PMID: 30361976

[ref47] Eskola M. Kos G. Elliott C. T. Hajšlová J. Mayar S. Krska R. (2020). Worldwide contamination of food-crops with mycotoxins: validity of the widely cited ‘FAO estimate’ of 25%. Crit. Rev. Food Sci. Nutr. 60, 2773–2789. doi: 10.1080/10408398.2019.1658570 31478403

[ref48] European Commission (2003). Regulation (EC) No 1831/2003 of the European Parliament and of the council of 22 September 2003 on additives for use in animal nutrition.

[ref49] European Commission (2006). Commission Regulation (EC) No. 1881/2006 of 19 December 2006 Setting maximum levels for certain contaminants in foodstuffs. Off. J. Eur. Union 2006, 364, 5–24.

[ref016] European Commission (2007). Available online at: https://eur-lex.europa.eu/legal-content/EN/TXT/PDF/?uri=CELEX:52007DC0354&from=EN

[ref50] European Commission (2010). Commission Regulation (EU) No. 165/2010 of 26 February 2010 Amending regulation (EC) no 1881/2006 setting maximum levels for certain contaminants in foodstuffs as regards aflatoxins. Off. J. Eur. Union 2010, 50, 8–12.

[ref51] European Commission (2015). Commission regulation (EU) 2015/786 of 19 May 2015 defining acceptability criteria for detoxification processes applied to products intended for animal feed as provided for in directive 2002/32/EC of the European Parliament and of the council.

[ref52] FAO (2022). Crop prospects and food situation. Available at: https://www.fao.org/3/cc0868en/cc0868en.pdf [Accessed September 19, 2022].

[ref53] FAOstat (2022). Available at: https://www.fao.org/faostat/en/ [Accessed November 26, 2022].

[ref015] FAOstat (2013). Available at: https://www.fao.org/3/i3107e/i3107e.PDF

[ref54] Farkas Z. Országh E. Engelhardt T. Csorba S. Kerekes K. Zentai A.. (2022). A systematic review of the efficacy of interventions to control aflatoxins in the dairy production chain—feed production and animal feeding interventions. Toxins 14:115. doi: 10.3390/toxins14020115 35202142 PMC8878089

[ref55] Fasoyin O. E. Wang B. Qiu M. Han X. Chung K. R. Wang S. (2018). Carbon catabolite repression gene *creA* regulates morphology, aflatoxin biosynthesis and virulence in *Aspergillus flavus*. Fungal Genet. Biol. 115, 41–51. doi: 10.1016/j.fgb.2018.04.008 29655909

[ref56] Fasoyin O. E. Yang K. Qiu M. Wang B. Wang S. Wang S. (2019). Regulation of morphology, aflatoxin production, and virulence of *Aspergillus flavus* by the major nitrogen regulatory gene *areA*. Toxins 11:718. doi: 10.3390/toxins11120718 31835504 PMC6950533

[ref57] Faustinelli P. C. Power I. L. Arias R. S. (2018). Detection of exogenous double-stranded RNA movement in *in vitro* peanut plants. Plant Biol. 20, 444–449. doi: 10.1111/plb.12703 29405546

[ref58] Ferrara M. Perrone G. Gallo A. (2022). Recent advances in biosynthesis and regulatory mechanisms of principal mycotoxins. Curr. Opin. Food Sci. doi: 10.1016/j.cofs.2022.100923

[ref59] Finotti E. Parroni A. Zaccaria M. Domin M. Momeni B. Fanelli C.. (2021). Aflatoxins are natural scavengers of reactive oxygen species. Sci. Rep. 11, 1–9. doi: 10.1038/s41598-021-95325-8 34362972 PMC8346536

[ref60] Fountain J. C. Scully B. T. Ni X. Kemerait R. C. Lee R. D. Chen Z. Y.. (2014). Environmental influences on maize-*Aspergillus flavus* interactions and aflatoxin production. Front. Microbiol. 5:40. doi: 10.3389/fmicb.2014.00040 24550905 PMC3913990

[ref61] Furukawa T. Sakuda S. (2019). Inhibition of aflatoxin production by paraquat and external superoxide dismutase in *Aspergillus flavus*. Toxins 11:107. doi: 10.3390/toxins11020107 30759855 PMC6409742

[ref62] Gamal M. Abou Zaid M. Abou Mourad I. K. Abd El Kareem H. Gomaa O. M. (2022). *Trichoderma viride* bioactive peptaibol induces apoptosis in *Aspergillus niger* infecting tilapia in fish farms. Aquaculture 547:737474. doi: 10.1016/j.aquaculture.2021.737474

[ref63] Georgianna D. R. Payne G. A. (2009). Genetic regulation of aflatoxin biosynthesis: from gene to genome. Fungal Genet. Biol. 46, 113–125. doi: 10.1016/j.fgb.2008.10.011 19010433

[ref64] Gilbert M. K. Majumdar R. Rajasekaran K. Chen Z. Y. Wei Q. Sickler C. M.. (2018). RNA interference-based silencing of the alpha-amylase (*amy1*) gene in *Aspergillus flavus* decreases fungal growth and aflatoxin production in maize kernels. Planta 247, 1465–1473. doi: 10.1007/s00425-018-2875-0 29541880

[ref65] Gil-Serna J. Vázquez C. Patiño B. (2020). Genetic regulation of aflatoxin, ochratoxin a, trichothecene, and fumonisin biosynthesis: a review. Int. Microbiol. 23, 89–96. doi: 10.1007/s10123-019-00084-2 31144067

[ref67] Gressel J. Polturak G. (2018). Suppressing aflatoxin biosynthesis is not a breakthrough if not useful. Pest Manag. Sci. 74, 17–21. doi: 10.1002/ps.4694 28762637

[ref68] Guan Y. Chen J. Nepovimova E. Long M. Wu W. Kuca K. (2021). Aflatoxin detoxification using microorganisms and enzymes. Toxins 13:46. doi: 10.1080/10408398.2021.2010647 33435382 PMC7827145

[ref69] Guan X. Zhao Y. Liu X. Shang B. Xing F. Zhou L.. (2019). The bZIP transcription factor Afap1 mediates the oxidative stress response and aflatoxin biosynthesis in *Aspergillus flavus*. Rev. Argent. Microbiol. 51, 292–301. doi: 10.1016/j.ram.2018.07.003 30905507

[ref70] Guo Y. Zhao L. Ma Q. Ji C. (2021). Novel strategies for degradation of aflatoxins in food and feed: a review. Food Res. Int. 140:109878. doi: 10.1016/j.foodres.2020.109878 33648196

[ref71] Heinl S. Hartinger D. Thamhesl M. Vekiru E. Krska R. Schatzmayr G.. (2010). Degradation of fumonisin B_1_ by the consecutive action of two bacterial enzymes. J. Biotechnol. 145, 120–129. doi: 10.1016/j.jbiotec.2009.11.004 19922747

[ref72] Hellegers P. (2022). Food security vulnerability due to trade dependencies on Russia and Ukraine. Food Secur., 1–8. doi: 10.1007/s12571-022-01306-8 PMC9304541 35891962

[ref73] Hojnik N. Modic M. Walsh J. L. Žigon D. Javornik U. Plavec J.. (2021). Unravelling the pathways of air plasma induced aflatoxin B_1_ degradation and detoxification. J. Hazard. Mater. 403:123593. doi: 10.1016/j.jhazmat.2020.123593 33264852

[ref74] Hong S. Y. Roze L. V. Linz J. E. (2013a). Oxidative stress-related transcription factors in the regulation of secondary metabolism. Toxins 5, 683–702. doi: 10.3390/toxins5040683 23598564 PMC3705287

[ref75] Hong S. Y. Roze L. V. Wee J. Linz J. E. (2013b). Evidence that a transcription factor regulatory network coordinates oxidative stress response and secondary metabolism in *Aspergilli*. Microbiol. Open 2, 144–160. doi: 10.1002/mbo3.63 PMC3584220 23281343

[ref76] Horky P. Skalickova S. Baholet D. Skladanka J. (2018). Nanoparticles as a solution for eliminating the risk of mycotoxins. Nano 8:727. doi: 10.3390/nano8090727 PMC6164963 30223519

[ref77] Hua S. S. T. Beck J. J. Sarreal S. B. L. Gee W. (2014). The major volatile compound 2-phenylethanol from the biocontrol yeast, *Pichia anomala*, inhibits growth and expression of aflatoxin biosynthetic genes of *Aspergillus flavus*. Mycotoxin Res. 30, 71–78. doi: 10.1007/s12550-014-0189-z 24504634

[ref78] International Agency for Research on Cancer (2002). Some traditional herbal medicines, some mycotoxins, naphthalene and styrene. Available at: http://monographs.iarc.fr/ENG/Monographs/vol82/volume82.pdf PMC4781602 12687954

[ref79] IPCC (2022). Climate change 2022: impacts, adaptation and vulnerability. Contribution of working group II to the sixth assessment report of the intergovernmental panel on climate change. eds. Pörtner H.-O. Roberts D. C. Tignor M. Poloczanska E. S. Mintenbeck K. Alegría A.. (Cambridge, UK and New York, NY, United States: Cambridge University Press), 3056.

[ref80] Isaschar-Ovdat S. Fishman A. (2018). Crosslinking of food proteins mediated by oxidative enzymes–a review. Trends Food Sci. Technol. 72, 134–143. doi: 10.1016/j.tifs.2017.12.011

[ref81] Jayashree T. Subramanyam C. (2000). Oxidative stress as a prerequisite for aflatoxin production by *Aspergillus parasiticus*. Free Radic. Biol. Med. 29, 981–985. doi: 10.1016/s0891-5849(00)00398-1 11084286

[ref82] Keller N. P. (2015). Translating biosynthetic gene clusters into fungal armor and weaponry. Nat. Chem. Biol. 11, 671–677. doi: 10.1038/nchembio.1897. 26284674 PMC4682562

[ref83] Keller N. P. Nesbitt C. Sarr B. Phillips T. D. Burow G. B. (1997). pH regulation of sterigmatocystin and aflatoxin biosynthesis in *Aspergillus* spp. Phytopathology 87, 643–648. doi: 10.1094/PHYTO.1997.87.6.643 18945083

[ref84] Khan R. Ghazali F. M. Mahyudin N. A. Samsudin N. I. P. (2021). Aflatoxin biosynthesis, genetic regulation, toxicity, and control strategies: a review. J. Fungi 7:606. doi: 10.3390/jof7080606 PMC8397101 34436145

[ref85] King T. Cole M. Farber J. M. Eisenbrand G. Zabaras D. Fox E. M.. (2017). Food safety for food security: relationship between global megatrends and developments in food safety. Trends Food Sci. Technol. 68, 160–175. doi: 10.1016/j.tifs.2017.08.014

[ref86] Kocsis B. Lee M. K. Yu J. H. Nagy T. Daróczi L. Batta G.. (2022). Functional analysis of the bZIP-type transcription factors AtfA and AtfB in *Aspergillus nidulans*. Front. Microbiol. 13:1003709. doi: 10.3389/fmicb.2022.1003709 36204617 PMC9530789

[ref87] Kolawole O. Meneely J. Greer B. Chevallier O. Jones D. S. Connolly L.. (2019). Comparative *in vitro* assessment of a range of commercial feed additives with multiple mycotoxin binding claims. Toxins 11:659. doi: 10.3390/toxins11110659 31726774 PMC6891808

[ref88] Kong Q. Chi C. Yu J. Shan S. Li Q. Guan B.. (2014). The inhibitory effect of *Bacillus megaterium* on aflatoxin and cyclopiazonic acid biosynthetic pathway gene expression in *Aspergillus flavus*. Appl. Environ. Microbiol. 98, 5161–5172. doi: 10.1007/s00253-014-5632-8 24652062

[ref89] Kos J. Janić Hajnal E. Šarić B. Jovanov P. Mandić A. Đuragić O.. (2018). Aflatoxins in maize harvested in the Republic of Serbia over the period 2012–2016. Food Addit. Contam. Part B 11, 246–255. doi: 10.1080/19393210.2018.1499675 30157711

[ref90] Kotta-Loizou I. Coutts R. H. A. (2017). Mycoviruses in aspergilli: a comprehensive review. Front. Microbiol. 8:1699. doi: 10.3389/fmicb.2017.01699 28932216 PMC5592211

[ref91] Kumar V. Bahuguna A. Ramalingam S. Dhakal G. Shim J. J. Kim M. (2022). Recent technological advances in mechanism, toxicity, and food perspectives of enzyme-mediated aflatoxin degradation. Crit. Rev. Food Sci. Nutr. 62, 5395–5412. doi: 10.1080/10408398.2021.2010647 34955062

[ref92] Kumar P. Mahato D. K. Kamle M. Mohanta T. K. Kang S. G. (2017). Aflatoxins: a global concern for food safety, human health and their management. Front. Microbiol. 7:2170. doi: 10.3389/fmicb.2016.02170 28144235 PMC5240007

[ref93] Lewis M. H. Carbone I. Luis J. M. Payne G. A. Bowen K. L. Hagan A. K.. (2019). Biocontrol strains differentially shift the genetic structure of indigenous soil populations of *Aspergillus flavus*. Front. Microbiol. 10:1738. doi: 10.3389/fmicb.2019.01738 31417528 PMC6685141

[ref94] Li S. Yao X. Wang X. Tian S. Zhang Y. (2022). Reactive molecular dynamics simulation on degradation of aflatoxin B_1_ by cold atmospheric plasmas. IFSET 80:103101. doi: 10.1016/j.ifset.2022.103101

[ref95] Lind A. L. Smith T. D. Saterlee T. Calvo A. M. Rokas A. (2016). Regulation of secondary metabolism by the velvet complex is temperature-responsive in *Aspergillus*. G3 Genes Genom. Genet. 6, 4023–4033. doi: 10.1128/aem.64.10.3718-3723.1998 PMC5144971 27694115

[ref96] Liu B. H. Chu F. S. (1998). Regulation of *aflR* and its product, AflR, associated with aflatoxin biosynthesis. Appl. Environ. Microbiol. 64, 3718–3723. doi: 10.1128/aem.64.10.3718-3723.1998 9758790 PMC106529

[ref05] Loi M. Fanelli F. Liuzzi V. C. Logrieco A. F. Mulè G. (2017). Mycotoxin biotransformation by native and commercial enzymes: present and future perspectives. Toxins 9:111. doi: 10.3390/toxins9040111 28338601 PMC5408185

[ref97] Loi M. Fanelli F. Zucca P. Liuzzi V. C. Quintieri L. Cimmarusti M. T.. (2016). Aflatoxin B_1_ and M_1_ degradation by Lac2 from *Pleurotus pulmonarius* and redox mediators. Toxins 8:245. doi: 10.3390/toxins8090245 27563923 PMC5037472

[ref98] Loi M. Quintieri L. De Angelis E. Monaci L. Logrieco A. F. Caputo L.. (2020a). Yield improvement of the Italian fresh Giuncata cheese by laccase–induced protein crosslink. Int. Dairy J. doi: 10.1016/j.idairyj.2019.104555

[ref99] Loi M. Quintieri L. Fanelli F. Caputo L. Mulè G. (2018). Application of a recombinant laccase-chlorogenic acid system in protein crosslink and antioxidant properties of the curd. Food Res. Int. 106, 763–770. doi: 10.1016/j.foodres.2018.01.050 29579985

[ref100] Loi M. Renaud J. B. Rosini E. Pollegioni L. Vignali E. Haidukowski M.. (2020b). Enzymatic transformation of aflatoxin B_1_ by Rh_DypB peroxidase and characterization of the reaction products. Chemosphere 250:126296. doi: 10.1016/j.chemosphere.2020.126296 32135437

[ref09] Loi M. Villani A. Paciolla F. Mulè G. Paciolla C. (2021). Challenges and Opportunities of Light-Emitting Diode (LED) as Key to Modulate Antioxidant Compounds in Plants. A Review. Antioxidants 10:42. doi: 10.3390/antiox10010042 PMC7824119 33396461

[ref101] Lu D. Tang S. Li Y. Cong Z. Zhang X. Wu S. (2021). Magnetic-propelled Janus yeast cell robots functionalized with metal-organic frameworks for mycotoxin decontamination. Micromachines 12:797. doi: 10.3390/mi12070797 34357207 PMC8307641

[ref102] Lv C. Jin J. Wang P. Dai X. Liu Y. Zheng M.. (2019). Interaction of water activity and temperature on the growth, gene expression and aflatoxin production by *Aspergillus flavus* on paddy and polished rice. Food Chem. 293, 472–478. doi: 10.1016/j.foodchem.2019.05.009 31151636

[ref103] Ma D. (2019). “Hybrid nanoparticles: an introduction,” in Noble metal-metal oxide hybrid nanoparticles (Duxford CB22 4QH, United Kindom: Woodhead Publishing), 3–6.

[ref104] Ma F. Cai X. Mao J. Yu L. Li P. (2021). Adsorptive removal of aflatoxin B_1_ from vegetable oils via novel adsorbents derived from a metal-organic framework. J. Hazard. Mater. 412:125170. doi: 10.1016/j.jhazmat.2021.125170 33951856

[ref105] Magzoub R. A. M. Yassin A. A. A. Abdel-Rahim A. M. Gubartallah E. A. Miskam M. Saad B.. (2019). Photocatalytic detoxification of aflatoxins in Sudanese peanut oil using immobilized titanium dioxide. Food Control 95, 206–214. doi: 10.1016/j.foodcont.2018.08.009

[ref106] Mahoney N. Molyneux R. J. (2004). Phytochemical inhibition of aflatoxigenicity in *Aspergillus flavus* by constituents of walnut (*Juglans regia*). J. Agric. Food Chem. 52, 1882–1889. doi: 10.1021/jf030812p 15053524

[ref107] Majumdar R. Lebar M. Mack B. Minocha R. Minocha S. Carter-Wientjes C.. (2018). The *Aspergillus flavus* spermidine synthase (*spds*) gene, is required for normal development, aflatoxin production, and pathogenesis during infection of maize kernels. Front. Plant Sci. 9:317. doi: 10.3389/fpls.2018.00317 29616053 PMC5870473

[ref108] Majumdar R. Rajasekaran K. Cary J. W. (2017). RNA interference (RNAi) as a potential tool for control of mycotoxin contamination in crop plants: concepts and considerations. Front. Plant Sci. 8:200. doi: 10.3389/fpls.2017.00200 28261252 PMC5306134

[ref109] Malhotra N. Lee J. S. Liman R. A. D. Ruallo J. M. S. Villaflores O. B. Ger T. R.. (2020). Potential toxicity of iron oxide magnetic nanoparticles: a review. Molecules 25:3159. doi: 10.3390/molecules25143159 32664325 PMC7397295

[ref110] Mandal R. Singh A. Pratap S. A. (2018). Recent developments in cold plasma decontamination technology in the food industry. Trends Food Sci. Technol. 80, 93–103. doi: 10.1016/j.tifs.2018.07.014

[ref111] Marshall H. Meneely J. P. Quinn B. Zhao Y. Bourke P Gilmore B. F. Elliott C. T. (2020). Novel decontamination approaches and their potential application for post-harvest aflatoxin control. Trends Food Sci. Technol., 106, 489–496. doi: 10.1016/j.tifs.2020.11.001

[ref112] Masanga J. O. Matheka J. M. Omer R. A. Ommeh S. C. Monda E. O. Alakonya A. E. (2015). Downregulation of transcription factor *aflR* in *Aspergillus flavus* confers reduction to aflatoxin accumulation in transgenic maize with alteration of host plant architecture. Plant Cell Rep. 34, 1379–1387. doi: 10.1007/s00299-015-1794-9 25895735

[ref113] Mauro A. Battilani P. Callicott K. A. Giorni P. Pietri A. Cotty P. J. (2013). Structure of an *Aspergillus flavus* population from maize kernels in northern Italy. Int. J. Food Microbiol. 162, 1–7. doi: 10.1016/j.ijfoodmicro.2012.12.021 23340386

[ref114] Mauro A. Garcia-Cela E. Pietri A. Cotty P. J. Battilani P. (2018). Biological control products for aflatoxin prevention in Italy: commercial field evaluation of atoxigenic *Aspergillus flavus* active ingredients. Toxins 10:30. doi: 10.3390/toxins10010030 29304008 PMC5793117

[ref115] McDonald T. Brown D. Keller N. P. Hammond T. M. (2005). RNA silencing of mycotoxin production in *Aspergillus* and *Fusarium* species. Mol. Plant-Microbe Interact. 18, 539–545. doi: 10.1094/MPMI-18-0539 15986923

[ref116] Medina A. Rodriguez A. Magan N. (2014). Effect of climate change on *Aspergillus flavus* and aflatoxin B_1_ production. Front. Microbiol. 5:348. doi: 10.3389/fmicb.2014.00348, PMID: 25101060 PMC4106010

[ref117] Montibus M. Pinson-Gadais L. Richard-Forget F. Barreau C. Ponts N. (2013). Coupling of transcriptional response to oxidative stress and secondary metabolism regulation in filamentous fungi. Crit. Rev. Microbiol. 41, 295–308. doi: 10.3109/1040841X.2013.829416 24041414

[ref118] Moore G. G. (2022). Practical considerations will ensure the continued success of pre-harvest biocontrol using non-aflatoxigenic *Aspergillus flavus* strains. Crit. Rev. Food Sci. Nutr. 62, 4208–4225. doi: 10.1080/10408398.2021.1873731 33506687

[ref119] Moore G. G. Lebar M. D. Carter-Wientjes C. H. Gilbert M. K. (2021). The potential role of fungal volatile organic compounds in *Aspergillus flavus* biocontrol efficacy. Biol. Control 160:104686. doi: 10.1016/j.biocontrol.2021.104686

[ref120] Moore G. G. Singh R. Horn B. W. Carbone I. (2009). Recombination and lineage-specific gene loss in the aflatoxin gene cluster of *Aspergillus flavus*. Mol. Ecol. 18, 4870–4887. doi: 10.1111/j.1365-294X.2009.04414.x 19895419

[ref121] Moral J. Garcia-Lopez M. T. Camiletti B. X. Jaime R. Michailides T. J. Bandyopadhyay R.. (2020). Present status and perspective on the future use of aflatoxin biocontrol products. Agronomy 10:491. doi: 10.3390/agronomy10040491

[ref122] Moretti A. Pascale M. Logrieco A. F. (2019). Mycotoxin risks under a climate change scenario in Europe. Trends Food Sci. Technol. 84, 38–40. doi: 10.1016/j.tifs.2018.03.008

[ref123] Murugesan P. Brunda D. K. Moses J. A. Anandharamakrishnan C. (2021). Photolytic and photocatalytic detoxification of mycotoxins in foods. Food Control 123:107748. doi: 10.1016/j.foodcont.2020.107748

[ref124] Nahle S. El Khoury A. Savvaidis I. Chokr A. Louka N. Atoui A. (2022). Detoxification approaches of mycotoxins: by microorganisms, biofilms and enzymes. Int. J. Food Contam. 9, 1–14. doi: 10.1186/s40550-022-00089-2

[ref125] Niehaus W. G. Jiang W. P. (1989). Nitrate induces enzymes of the mannitol cycle and suppresses versicolorin synthesis in *Aspergillus parasiticus*. Mycopathologia 107, 131–137. 2615792 10.1007/BF00707550

[ref126] Niño-Sánchez J. Chen L. H. De Souza J. T. Mosquera S. Stergiopoulos I. (2021). Targeted delivery of gene silencing in fungi using genetically engineered bacteria. J. Fungi 7:125. doi: 10.3390/jof7020125, PMID: 33572197 PMC7914413

[ref127] O’Brian G. R. Georgianna D. R. Wilkinson J. R. Yu J. Abbas H. K. Bhatnagar D.. (2007). The effect of elevated temperature on gene transcription and aflatoxin biosynthesis. Mycologia 99, 232–239. doi: 10.3852/mycologia.99.2.232 17682776

[ref128] Ola O. T. Ogedengbe O. O. Raji T. M. Eze B. Chama M. Ilori O. N.. (2022). Aflatoxin biocontrol effectiveness in the real world—private sector-led efforts to manage aflatoxins in Nigeria through biocontrol-centered strategies. Front. Microbiol. 13:977789. doi: 10.3389/fmicb.2022.977789 36118233 PMC9478371

[ref129] Omolehin O. Raruang Y. Hu D. Han Z. Q. Wei Q. Wang K.. (2021). Resistance to aflatoxin accumulation in maize mediated by host-induced silencing of the *Aspergillus flavus* alkaline protease (*alk*) gene. J. Fungi 7:904. doi: 10.3390/jof7110904 PMC8622731 34829193

[ref130] Ortega-Beltran A. Agbetiameh D. Atehnkeng J. Falade T. D. Bandyopadhyay R. (2021). Does use of Atoxigenic biocontrol products to mitigate aflatoxin in maize increase fumonisin content in grains? Plant Dis. 105, 2196–2201. doi: 10.1094/PDIS-07-20-1447-RE 33210967

[ref131] Ortega-Beltran A. Bandyopadhyay R. (2021). Contributions of integrated aflatoxin management strategies to achieve the sustainable development goals in various African countries. Glob. Food Sec. 30:100559. doi: 10.1016/j.gfs.2021.100559

[ref132] Peles F. Sipos P. Kovács S. Győri Z. Pócsi I. Pusztahelyi T. (2021). Biological control and mitigation of aflatoxin contamination in commodities. Toxins 13:104. doi: 10.3390/toxins13020104 33535580 PMC7912779

[ref133] Pennerman K. K. Gonzalez J. Chenoweth L. R. Bennett J. W. Yin G. Hua S. S. T. (2018). Biocontrol strain *Aspergillus flavus* WRRL 1519 has differences in chromosomal organization and an increased number of transposon-like elements compared to other strains. MGG 293, 1507–1522. doi: 10.1007/s00438-018-1474-x 30099586

[ref134] Pérez-Gómez E. O. García-Rosales G. Longoria-Gándara L. C. Gómez-Vilchis J. C. (2022). Obtention of biochar-Ca nanoparticles using *citrus tangerina*: a morphological, surface and study remotion of aflatoxin AFB_1_. J. Hazard. Mater. 424:127339. doi: 10.1016/j.jhazmat.2021.127339 34879555

[ref135] Pfannenstiel B. T. Greco C. Sukowaty A. T. Keller N. P. (2018). The epigenetic reader SntB regulates secondary metabolism, development and global histone modifications in *Aspergillus flavus*. Fungal Genet. Biol. 120, 9–18. doi: 10.1016/j.fgb.2018.08.004 30130575 PMC6215504

[ref136] Pfliegler W. P. Pócsi I. Győri Z. Pusztahelyi T. (2020). The aspergilli and their mycotoxins: metabolic interactions with plants and the soil biota. Front. Microbiol. 10:2921. doi: 10.3389/fmicb.2019.02921 32117074 PMC7029702

[ref137] Piotrowska M. (2021). Microbiological decontamination of mycotoxins: opportunities and limitations. Toxins 13:819. doi: 10.3390/toxins13110819, PMID: 34822603 PMC8619243

[ref138] Power I. L. Faustinelli P. C. Orner V. A. Sobolev V. S. Arias R. S. (2020). Analysis of small RNA populations generated in peanut leaves after exogenous application of dsRNA and dsDNA targeting aflatoxin synthesis genes. Sci. Rep. 10, 1–12. doi: 10.1038/s41598-020-70618-6 32796886 PMC7427784

[ref139] Price M. S. Conners S. B. Tachdjian S. Kelly R. M. Payne G. A. (2005). Aflatoxin conducive and non-conducive growth conditions reveal new gene associations with aflatoxin production. Fungal Genet. Biol. 42, 506–518. doi: 10.1016/j.fgb.2005.03.009 15878831

[ref140] Qin X. Xin Y. Zou J. Su X. Wang X. Wang Y.. (2021). Efficient degradation of aflatoxin B_1_ and zearalenone by laccase-like multicopper oxidase from *Streptomyces thermocarboxydus* in the presence of mediators. Toxins 13:754. doi: 10.3390/toxins13110754 34822538 PMC8621583

[ref141] Ráduly Z. Szabó L. Madar A. Pócsi I. Csernoch L. (2020). Toxicological and medical aspects of *Aspergillus*-derived mycotoxins entering the feed and food chain. Front. Microbiol. 10:2908. doi: 10.3389/fmicb.2019.02908 31998250 PMC6962185

[ref142] Rapid Alert System for Food and Feed (2021). Annual Report. Available at: https://food.ec.europa.eu/system/files/2022-07/acn_annual-report_2021-final.pdf [Accessed November 26, 2022].

[ref143] Rapid Alert System for Food and Feed (2022). Portal Database. Available at: https://webgate.ec.europa.eu/rasff-window/portal/ [Accessed August 3, 2022].

[ref144] Raruang Y. Omolehin O. Hu D. Wei Q. Han Z. Q. Rajasekaran K.. (2020). Host induced gene silencing targeting *Aspergillus flavus* *aflM* reduced aflatoxin contamination in transgenic maize under field conditions. Front. Microbiol. 11:754. doi: 10.3389/fmicb.2020.00754 32411110 PMC7201132

[ref145] Regulation (EU) 2021/363. Commission Implementing Regulation (EU) 2021/363 of 26 February 2021. Concerning the authorisation of a preparation of fumonisin esterase produced by *Komagataella phaffii* DSM 32159 as a feed additive for all animal species (text with EEA relevance) C/2021/1140.

[ref146] Ren X. Branà M. T. Haidukowski M. Gallo A. Zhang Q. Logrieco A. F.. (2022). Potential of *trichoderma* spp. for biocontrol of aflatoxin-producing *Aspergillus flavus*. Toxins 14:86. doi: 10.3390/toxins14020086 35202114 PMC8875375

[ref147] Ren Y. Jin J. Zheng M. Yang Q. Xing F. (2020). Ethanol inhibits aflatoxin B_1_ biosynthesis in *Aspergillus flavus* by up-regulating oxidative stress-related genes. Front. Microbiol. 10:2946. doi: 10.3389/fmicb.2019.02946 32010073 PMC6978751

[ref148] Ren Z. Luo J. Wan Y. (2019). Enzyme-like metal–organic frameworks in polymeric membranes for efficient removal of aflatoxin B_1_. ACS Appl. Mater. Interfaces 11, 30542–30550. doi: 10.1021/acsami.9b08011 31362494

[ref149] Ren X. Zhang Q. Zhang W. Mao J. Li P. (2020). Control of aflatoxigenic molds by antagonistic microorganisms: inhibitory behaviors, bioactive compounds, related mechanisms, and influencing factors. Toxins 12:24. doi: 10.3390/toxins12010024 31906282 PMC7020460

[ref150] Reverberi M. Fabbri A. A. Zjalic S. Ricelli A. Punelli F. Fanelli C. (2005). Antioxidant enzymes stimulation in *Aspergillus parasiticus* by *Lentinula edodes* inhibits aflatoxin production. Appl. Microbiol. Biotechnol. 69, 207–215. doi: 10.1007/s00253-005-1979-1 15838675

[ref151] Reverberi M. Ricelli A. Zjalic S. Fabbri A. A. Fanelli C. (2010). Natural functions of mycotoxins and control of their biosynthesis in fungi. Appl. Microbiol. Biotechnol. 87, 899–911. doi: 10.1007/s00253-010-2657-5 20495914

[ref152] Reverberi M. Zjalic S. Ricelli A. Fabbri A. A. Fanelli C. (2006). Oxidant/antioxidant balance in *Aspergillus parasiticus* affects aflatoxin biosynthesis. Mycotoxin Res. 22, 39–47. doi: 10.1007/BF02954556 23605500

[ref153] Reverberi M. Zjalic S. Ricelli A. Punelli F. Camera E. Fabbri C.. (2008). Modulation of antioxidant defence in *Aspergillus parasiticus* is involved in aflatoxin biosynthesis: a role for Ap*yapA* gene. Eukaryot. Cell 7, 988–1000. doi: 10.1128/EC.00228-07 18441122 PMC2446656

[ref08] Rifna E. J. Ramanan K. R. Mahendran R. (2019). Emerging technology applications for improving seed germination. Trends in Food Science & Technology 86, 95–108. doi: 10.1016/j.tifs.2019.02.029

[ref154] Roze L. V. Chanda A. Wee J. Awad D. Linz J. E. (2011). Stress-related transcription factor AtfB integrates secondary metabolism with oxidative stress response in Aspergilli. J. Biol. Chem. 286, 35137–35148. doi: 10.1074/jbc.M111.253468 21808056 PMC3186425

[ref155] Rushing B. R. Selim M. I. (2019). Aflatoxin B_1_: a review on metabolism, toxicity, occurrence in food, occupational exposure, and detoxification methods. Food Chem. Toxicol. 124, 81–100. doi: 10.1016/j.fct.2018.11.047 30468841

[ref156] Safari N. Mirabzadeh Ardakani M. Hemmati R. Parroni A. Beccaccioli M. Reverberi M. (2020). The potential of plant-based bioactive compounds on inhibition of aflatoxin B_1_ biosynthesis and down-regulation of *aflR, aflM* and *aflP* genes. Antibiotics 9:728. doi: 10.3390/antibiotics9110728 33113979 PMC7690750

[ref157] Sarmast E. Fallah A. A. Jafari T. Khaneghah A. M. (2021). Occurrence and fate of mycotoxins in cereals and cereal-based products: a narrative review of systematic reviews and meta-analyses studies. Curr. Opin. Food Sci. 39, 68–75. doi: 10.1016/j.cofs.2020.12.013

[ref158] Sarrocco S. Mauro A. Battilani P. (2019). Use of competitive filamentous fungi as an alternative approach for mycotoxin risk reduction in staple cereals: state of art and future perspectives. Toxins 11:701. doi: 10.3390/toxins11120701 31810316 PMC6950288

[ref159] Schmidt F. R. (2009). The RNA interference-virus interplay: tools of nature for gene modulation, morphogenesis, evolution and a possible mean for aflatoxin control. Appl. Microbiol. Biotechnol. 83, 611–615. doi: 10.1007/s00253-009-2007-7 19466405

[ref160] Schmidt-Heydt M. Abdel-Hadi A. Magan N. Geisen R. (2009). Complex regulation of the aflatoxin biosynthesis gene cluster of *Aspergillus flavus* in relation to various combinations of water activity and temperature. Int. J. Food Microbiol. 135, 231–237. doi: 10.1016/j.ijfoodmicro.2009.07.026 19699547

[ref161] Schmidt-Heydt M. Rüfer C. E. Abdel-Hadi A. Magan N. Geisen R. (2010). The production of aflatoxin B_1_ or G_1_ by *Aspergillus parasiticus* at various combinations of temperature and water activity is related to the ratio of *aflS* to *aflR* expression. Mycotox. Res. 26, 241–246. doi: 10.1007/s12550-010-0062-7 23605486

[ref162] Shantha T. Murthy V. S. (1981). Influence of tricarboxylic acid cycle intermediates and related metabolites on the biosynthesis of aflatoxin by resting cells of *Aspergillus flavus*. Appl. Environ. Microbiol. 42, 758–761. doi: 10.1128/aem.42.5.758-761.1981 6797348 PMC244103

[ref011] Shen M. H. Singh R. K. (2021). Effect of rotating peanuts on aflatoxin detoxification by ultraviolet C light and irradiation uniformity evaluated by AgCl-based dosimeter. Food Control 120:107533. doi: 10.1016/j.foodcont.2020.107533

[ref163] Shen M. H. Singh R. K. (2022). Effective UV wavelength range for increasing aflatoxins reduction and decreasing oil deterioration in contaminated peanuts. Food Res. Int. 154:111016. doi: 10.1016/j.foodres.2022.111016 35337575

[ref164] Shimizu K. Hicks J. K. Huang T. P. Keller N. P. (2003). Pka, Ras and RGS protein interactions regulate activity of AflR, a Zn(II)2Cys6 transcription factor in *Aspergillus nidulans*. Genetics 165, 1095–1104. doi: 10.1093/genetics/165.3.1095 14668367 PMC1462812

[ref165] Shimizu K. Keller N. P. (2001). Genetic involvement of a cAMP-dependent protein kinase in a G protein signaling pathway regulating morphological and chemical transitions in *Aspergillus nidulans*. Genetics 157, 591–600. doi: 10.1093/genetics/157.2.591 11156981 PMC1461531

[ref166] Sipos P. Peles F. Brassó D. L. Béri B. Pusztahelyi T. Pócsi I.. (2021). Physical and chemical methods for reduction in aflatoxin content of feed and food. Toxins 13:204. doi: 10.3390/toxins13030204 33808964 PMC7999035

[ref167] Solomon S. Quin D. Manning M. Alley R. B. Berstein T. Bindoff N. I.. (2007). “Technical summary,” in Climate change 2007: The physical science basis. Contribution of working group 1 to the fourth assessment report of the intergovernmental panel on climate change. eds. Solomon S. Quin D. Manning M. Chen Z. Marquis M. Averyt K. B. Tignor M. Miller H. L. (Cambridge, NY: Cambridge University Press), 996.

[ref168] Song C. Qin J. (2022). High-performance fabricated nano-adsorbents as emerging approach for removal of mycotoxins: a review. Int. J. Food Sci. 57, 5781–5789. doi: 10.1111/ijfs.15953

[ref169] Song C. Yang J. Wang Y. Ding G. Guo L. Qin J. (2022). Mechanisms and transformed products of aflatoxin B_1_ degradation under multiple treatments: a review. Crit. Rev. Food Sci. Nutr. 1–13. doi: 10.1080/10408398.2022.2121910 36102160

[ref170] Sun S. Zhao R. Xie Y. Liu Y. (2019). Photocatalytic degradation of aflatoxin B_1_ by activated carbon supported TiO2 catalyst. Food Control 100, 183–188. doi: 10.1016/j.foodcont.2019.01.014

[ref171] Szabó Z. Pákozdi K. Murvai K. Pusztahelyi T. Kecskeméti Á. Gáspár A.. (2020). *FvatfA* regulates growth, stress tolerance as well as mycotoxin and pigment productions in *Fusarium verticillioides*. Appl. Microbiol. Biotechnol. 104, 7879–7899. doi: 10.1007/s00253-020-10717-6 32719911 PMC7447684

[ref172] Thakare D. Zhang J. Wing R. A. Cotty P. J. Schmidt M. A. (2017). Aflatoxin-free transgenic maize using host-induced gene silencing. Sci. Adv. 3:e1602382. doi: 10.1126/sciadv.1602382 28345051 PMC5345927

[ref173] Tilburn J. Sarkar S. Widdick D. A. Espeso E. A. Orejas M. Mungroo J.. (1995). The *Aspergillus* PacC zinc-finger transcription factor mediates regulation of both acid-expressed and alkaline-expressed genes by ambient pH. EMBO J. 14, 779–790. doi: 10.1002/j.1460-2075.1995.tb07056.x 7882981 PMC398143

[ref174] Tsitsigiannis D. I. Keller N. P. (2006). Oxylipins act as determinants of natural product biosynthesis and seed colonization in *Aspergillus nidulans*. Mol. Microbiol. 59, 882–892. doi: 10.1111/j.1365-2958.2005.05000.x 16420358

[ref175] Tsitsigiannis D. I. Keller N. P. (2007). Oxylipins as developmental and host–fungal communication signals. Trends Microbiol. 15, 109–118. doi: 10.1016/j.tim.2007.01.005, PMID: 17276068

[ref176] Tsitsigiannis D. I. Kunze S. Willis D. K. Feussner I. Keller N. P. (2005). *Aspergillus* infection inhibits the expression of peanut 13 S -HPODE-forming seed lipoxygenases. MPMI 18, 1081–1089. doi: 10.1094/MPMI-18-1081 16255247

[ref177] Van der Fels-Klerx H. J. Vermeulen L. C. Gavai A. K. Liu C. (2019). Climate change impacts on aflatoxin B_1_ in maize and aflatoxin M_1_ in milk: a case study of maize grown in Eastern Europe and imported to the Netherlands. PLoS One 14:e0218956. doi: 10.1371/journal.pone.0218956, PMID: 31247003 PMC6597076

[ref014] Wang P. Chang P.-K. Kong Q. Shan S. Wei Q. (2019). Comparison of aflatoxin production of *Aspergillus flavus* at different temperatures and media: Proteome analysis based on TMT. Int J Food Microbiol. 16:108313. doi: 10.1016/j.ijfoodmicro.2019.108313 31476580

[ref178] Wang Y. Chen Y. Jiang L. Huang H. (2022). Improvement of the enzymatic detoxification activity towards mycotoxins through structure-based engineering. Biotechnol. Adv. 107927. doi: 10.1016/j.biotechadv.2022.107927 35182727

[ref179] Wang H. Lei Y. Yan L. Cheng K. Dai X. Wan L.. (2015). Deep sequencing analysis of transcriptomes in *Aspergillus flavus* in response to resveratrol. BMC Microbiol. 15, 1–14. doi: 10.1186/s12866-015-0513-6 26420172 PMC4589122

[ref180] Wang X. Zha W. Liang L. Fasoyin O. E. Wu L. Wang S. (2020). The bZIP transcription factor AflRsmA regulates aflatoxin B_1_ biosynthesis, oxidative stress response and sclerotium formation in *Aspergillus flavus*. Toxins 12:271. doi: 10.3390/toxins12040271 32340099 PMC7232220

[ref181] Weaver M. A. Mack B. M. Gilbert M. K. (2019). Genome sequences of 20 georeferenced *Aspergillus flavus* isolates. Microbiol. Resour. Announc. 8:e01718. doi: 10.1128/MRA.01718-18 30938332 PMC6424216

[ref182] Wee J. Hong S.-Y. Roze L. V. Day D. M. Chanda A. Linz J. E. (2017). The fungal bZIP transcription factor AtfB controls virulence-associated processes in *Aspergillus parasiticus*. Toxins 9:287. doi: 10.3390/toxins9090287 28926946 PMC5618220

[ref183] Wei J. Wu X. Wu C. Hou F. Wu L. Huang H. (2022). Metal-organic frameworks with peroxidase-like activity for efficient removal of aflatoxin B_1_. Food Chem. 378:132037. doi: 10.1016/j.foodchem.2021.132037, PMID: 35045371

[ref184] Woo S. L. Ruocco M. Vinale F. Nigro M. Marra R. Lombardi N.. (2014). *Trichoderma*-based products and their widespread use in agriculture. Open Mycol. J. 8, 71–126. doi: 10.2174/1874437001408010071

[ref185] Wu F. (2022). Mycotoxin risks are lower in biotech corn. Curr. Opin. Biotechnol. 78:102792. doi: 10.1016/j.copbio.2022.102792 36088737

[ref186] Wu Y. Cheng J. H. Sun D. W. (2021). Blocking and degradation of aflatoxins by cold plasma treatments: applications and mechanisms. Trends Food Sci. Technol. 109, 647–661. doi: 10.1016/j.tifs.2021.01.053

[ref187] Wu Q. Fan J. Chen X. Zhu Z. Luo J. Wan Y. (2020). Sandwich structured membrane adsorber with metal organic frameworks for aflatoxin B_1_ removal. Sep. Purif. Technol. 246:116907. doi: 10.1016/j.seppur.2020.116907

[ref188] Wu F. Guclu H. (2012). Aflatoxin regulations in a network of global maize trade. PLoS One 7:e45151. doi: 10.1371/journal.pone.0045151 23049773 PMC3458029

[ref190] Xu D. Peng S. Guo R. Yao L. Mo H. Li H.. (2021). EGCG alleviates oxidative stress and inhibits aflatoxin B_1_ biosynthesis via MAPK signaling pathway. Toxins 13:693. doi: 10.3390/toxins13100693 34678986 PMC8539566

[ref191] Yang P. Xiao W. Lu S. Jiang S. Zheng Z. Zhang D.. (2021). Recombinant expression of *Trametes versicolor* aflatoxin B_1_-degrading enzyme (TV-AFB_1_D) in engineering *Pichia pastoris* GS115 and application in AFB_1_ degradation in AFB_1_-contaminated peanuts. Toxins 13:349. doi: 10.3390/toxins13050349 34068167 PMC8153001

[ref192] Yin W. B. Amaike S. Wohlbach D. J. Gasch A. P. Chiang Y. M. Wang C. C.. (2012). An *Aspergillus nidulans* bZIP response pathway hardwired for defensive secondary metabolism operates through *aflR*. Mol. Microbiol. 83, 1024–1034. doi: 10.1111/j.1365-2958.2012.07986.x, PMID: 22283524 PMC3288630

[ref193] Yin W. B. Reinke A. W. Szilágyi M. Emri T. Chiang Y. M. Keating A. E.. (2013). bZIP transcription factors affecting secondary metabolism, sexual development and stress responses in *Aspergillus nidulans*. Microbiology 159, 77–88. doi: 10.1099/mic.0.063370-0 23154967 PMC3542729

[ref194] Yu J. (2012). Current understanding on aflatoxin biosynthesis and future perspective in reducing aflatoxin contamination. Toxins 4, 1024–1057. doi: 10.3390/toxins4111024 23202305 PMC3509697

[ref195] Yu J.-H. Butchko R. E. Fernandes M. Keller N. Leonard T. Adams T. (1996). Conservation of structure and function of the aflatoxin regulatory gene *aflR* from *Aspergillus nidulans* and *A. flavus*. Curr. Genet. 29, 549–555. doi: 10.1007/BF02426959 8662194

[ref196] Yu J. Chang P. Ehrlich K. C. Cary J. W. Bhatnagar D. Cleveland T. E.. (2004). Clustered pathway genes in aflatoxin biosynthesis. Appl. Environ. Microbiol. 70, 1253–1262. doi: 10.1128/AEM.70.3.1253-1262.2004 15006741 PMC368384

[ref197] Yu J. Woloshuk C. P. Bhatnagar D. Cleveland T. E. (2000). Cloning and characterization of *avfA* and *omtB* genes involved in aflatoxin biosynthesis in three *Aspergillus* species. Gene 248, 157–167. doi: 10.1016/s0378-1119(00)00126-8 10806361

[ref198] Yunes N. Oliveira R. C. Reis T. A. Baquião A. C. Rocha L. O. Correa B. (2020). Effect of temperature on growth, gene expression, and aflatoxin production by *Aspergillus nomius* isolated from Brazil nuts. Mycotoxin Res. 36, 173–180. doi: 10.1007/s12550-019-00380-w 31828531

[ref199] Zhang F. Guo Z. Zhong H. Wang S. Yang W. Liu Y.. (2014). RNA-Seq-based transcriptome analysis of aflatoxigenic *Aspergillus flavus* in response to water activity. Toxins 6, 3187–3207. doi: 10.3390/toxins6113187 25421810 PMC4247253

[ref200] Zhang X. Li G. Wu D. Liu J. Wu Y. (2020). Recent advances on emerging nanomaterials for controlling the mycotoxin contamination: from detection to elimination. Food Front. 1, 360–381. doi: 10.1002/fft2.42

[ref201] Zhang C. Selvaraj J. N. Yang Q. Liu Y. (2017). A survey of aflatoxin-producing *Aspergillus* sp. from peanut field soils in four agroecological zones of China. Toxins 9:E40. doi: 10.3390/toxins9010040 PMC5308272 28117685

[ref202] Zhao Q. Pei H. Zhou X. Zhao K. Yu M. Han G.. (2022). Systematic characterization of bZIP transcription factors required for development and aflatoxin generation by high-throughput gene knockout in *Aspergillus flavus*. J Fungi 8:356. doi: 10.3390/jof8040356 PMC9031554 35448587

[ref203] Zhao X. Zhi Q. Q. Li J. Y. Keller N. P. He Z. M. (2018). The antioxidant gallic acid inhibits aflatoxin formation in *Aspergillus flavus* by modulating transcription factors FarB and CreA. Toxins 10:270. doi: 10.3390/toxins10070270 29970790 PMC6071284

[ref204] Zhu Y. Hassan Y. I. Lepp D. Shao S. Zhou T. (2017). Strategies and methodologies for developing microbial detoxification systems to mitigate mycotoxins. Toxins 9:130. doi: 10.3390/toxins9040130 28387743 PMC5408204

[ref205] Zhu Y. Hassan Y. I. Watts C. Zhou T. (2016). Innovative technologies for the mitigation of mycotoxins in animal feed and ingredients—A review of recent patents. Anim. Feed Sci. Technol. 216, 19–29. doi: 10.1016/j.anifeedsci.2016.03.030

[ref206] Zjalic S. Reverberi M. Ricelli A. Granito V. M. Fanelli C. Fabbri A. A. (2006). *Trametes versicolor*: a possible tool for aflatoxin control. Int. J. Food Microbiol. 107, 243–249. doi: 10.1016/j.ijfoodmicro.2005.10.003 16337299

